# Peptidomimetic antibiotics disrupt the lipopolysaccharide transport bridge of drug-resistant Enterobacteriaceae

**DOI:** 10.1126/sciadv.adg3683

**Published:** 2023-05-24

**Authors:** Matthias Schuster, Emile Brabet, Kathryn K. Oi, Nicolas Desjonquères, Kerstin Moehle, Karen Le Poupon, Sophie Hell, Stéphane Gable, Virginie Rithié, Séverine Dillinger, Peter Zbinden, Anatol Luther, Claudia Li, Sarah Stiegeler, Carolin D’Arco, Hans Locher, Tobias Remus, Selena DiMaio, Paola Motta, Achim Wach, Françoise Jung, Grégory Upert, Daniel Obrecht, Mohammed Benghezal, Oliver Zerbe

**Affiliations:** ^1^ University of Zürich, Winterthurerstrasse 190, CH-8057 Zürich, Switzerland.; ^2^ Spexis AG, Hegenheimermattweg 125, CH-4112 Allschwil, Switzerland.

## Abstract

The rise of antimicrobial resistance poses a substantial threat to our health system, and, hence, development of drugs against novel targets is urgently needed. The natural peptide thanatin kills Gram-negative bacteria by targeting proteins of the lipopolysaccharide transport (Lpt) machinery. Using the thanatin scaffold together with phenotypic medicinal chemistry, structural data, and a target-focused approach, we developed antimicrobial peptides with drug-like properties. They exhibit potent activity against Enterobacteriaceae both in vitro and in vivo while eliciting low frequencies of resistance. We show that the peptides bind LptA of both wild-type and thanatin-resistant *Escherichia coli* and *Klebsiella pneumoniae* strains with low-nanomolar affinities. Mode of action studies revealed that the antimicrobial activity involves the specific disruption of the Lpt periplasmic protein bridge.

## Teaser

Peptides with druglike properties and activity against Enterobacteriaceae are described together with their mode of action.

## INTRODUCTION

In 2019, an estimated 5 million deaths were associated with antimicrobial resistance (AMR) worldwide (*1*). World Health Organization priority 1 pathogens (*Escherichia coli*, *Staphylococcus aureus*, *Klebsiella pneumoniae*, *Streptococcus pneumoniae*, *Acinetobacter baumannii*, and *Pseudomonas aeruginosa*) account for the majority of the antibiotic-resistant infections. Thus, there is a critical and growing need to replenish the arsenal of standard-of-care (SoC) antibiotics to bring new solutions to AMR bacterial infections (*2*, *3*), particularly against carbapenem-resistant Enterobacteriaceae (CRE) (*3*) that cause difficult-to-treat nosocomial infections (*4*).

**Fig. 1.**
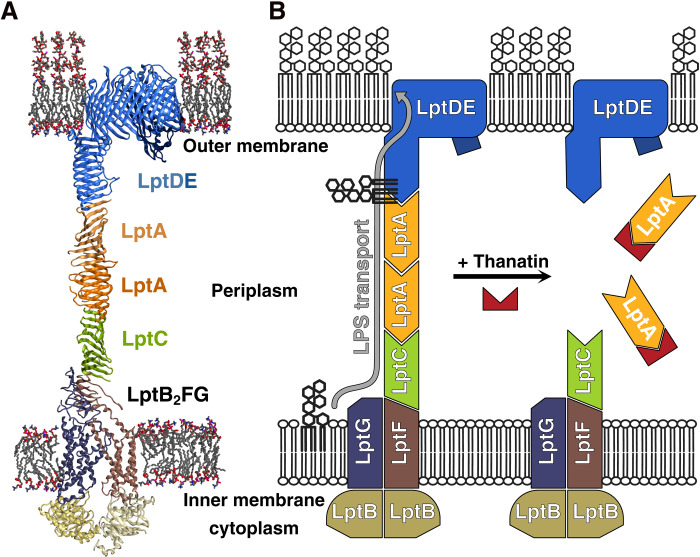
Structural model of the Lpt machinery. (**A**) Protein assembly of the Lpt in Gram-negative bacteria. (**B**) Schematic representation of the postulated mode of action based on this work: Thanatin inhibits the LptA-LptA and LptC-LptA protein-protein interactions and disassembles the Lpt periplasmic protein bridge. Note that two LptA molecules are depicted although the exact number is unknown.

One way of reducing the threat of AMR organisms is to develop antibiotics against novel targets. Recently, naturally occurring peptides were proposed to interfere with the function of proteins constituting the lipopolysaccharide (LPS) transport (Lpt) machinery or the β-barrel assembly machinery (*5*–*8*) and represent an untapped target source. LPS transport to the bacterial outer membrane is accomplished by a system of seven proteins, called Lpt proteins A to G (Fig. 1) (*9*–*11*). The periplasmic protein bridge is composed of one or more LptA molecules anchored to LptC and LptD at the inner and outer membranes, respectively. Dysregulation of lipid A biosynthesis and LPS transport results in lethal LPS accumulation at the inner membrane (*5*, *12*–*17*).

Thanatin, a 21–amino acid defense peptide isolated from the gut of the hemipteran insect *Podisus maculiventris* (*18*), exhibits broad-spectrum antimicrobial activity. Recent mechanism of action studies revealed that thanatin binds to LPS and with high affinity to the periplasmic proteins LptA and LptD (*16*, *19*). We hypothesized that thanatin mainly acts as a competitive inhibitor of the protein-protein interactions mediating the Lpt bridge assembly (Fig. 1), thereby inhibiting LPS transport across the periplasm. Thanatin, however, is not a suitable drug candidate for further development due to poor drug-like properties and rapid emergence of resistance (*16*).

Here, we introduce thanatin-derived synthetic macrocyclic peptides found after a substantial medicinal chemistry effort. The novel lead candidates show potent antimicrobial activity against CRE in vitro and in mouse infection models, favorable absorption, distribution, metabolism, and excretion (ADME) and a good safety profile. Compared to SoC antibiotics, they are also active against multidrug-resistant (MDR) and extensively drug-resistant (XDR) Enterobacteriaceae and have a low propensity to select for resistance. Furthermore, we show that these novel antibiotics bind in the low-nanomolar range to LptA and to a mutant of LptA conferring resistance to thanatin. These observations provide direct biophysical evidence of the disruption of the Lpt protein bridge and validate this macromolecular structure as a viable antibiotic target to tackle AMR.

## RESULTS

**Fig. 2.**
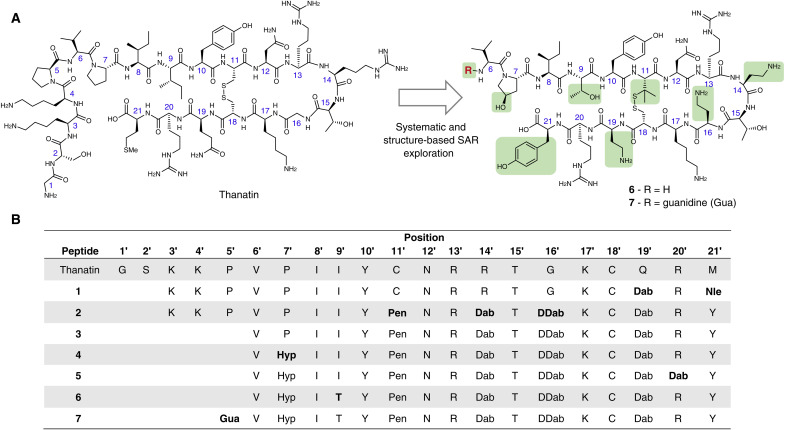
Thanatin and optimized analogs. (**A**) Chemical structures of thanatin and of the new antibiotic peptide analogs **6** and **7 **as modified during the structure-activity relationship (SAR) exploration. Structural modifications implemented during the medicinal chemistry optimization are highlighted in green. (**B**) Amino acid sequences (one letter code) of the described peptides. Thanatin and thanatin analogs residues are marked with an apostrophe. DDab, d-2,3-diaminobutyric acid.

Inspired by thanatin’s novel mechanism of action (*16*, *17*, *19*) and low propensity to permeabilize biological membranes (*16*, *18*, *20*), we sought to optimize thanatin-derived macrocyclic peptides to yield potent antibiotics with drug-like properties. The original report on the discovery of thanatin described a set of N-terminal truncated thanatin analogs with decreased antifungal activity but conserved antimicrobial activity (*18*). Particularly intrigued by the increase in selectivity toward Gram-negative bacteria, we followed a similar strategy and synthesized a first set of analogs shortened by the two N-terminal residues. The 19-mer peptide **1** (Fig. 2) shows increased bactericidal activity against our panel of *E. coli* and *K. pneumoniae* strains (Table 1). In contrast, truncation at the peptide C terminus led to a complete loss of antimicrobial activity (*18*).

**Table 1.** MIC values of investigated compounds and references against different parent strains and clones harboring the LtpA^Q62L^ mutation. MIC values (in micrograms per milliliter) were determined using the CLSI method. NDM-1, strain expressing the New Delhi metallo-β-lactamase 1 enzyme; CST-R, colistin-resistant strain; **6**-enant., all-(d)-**6** enantiomer; CAZ-AVI, ceftazidime-avibactam; n.t., not tested.

	*E. coli* ATCC 25922		*K. pneumoniae* ATCC 43816		*K. pneumoniae* NCTC 13443 (NDM-1)		*K. pneumoniae* 968733 (CST-R)
	Parent	Q62L mutant	Parent	Q62L mutant	Parent	Q62L mutant	Parent
Thanatin	1	>8	2	>8	8	>8	2
**1**	0.063	>8	0.25	>8	0.5	>8	0.25
**2**	0.031	2	0.125	2	0.5	>8	0.125
**3**	0.063	1	n.t.	n.t.	n.t.	n.t.	0.25
**4**	0.063	0.25	0.25	0.25	1	4	0.5
**5**	0.063	0.25	0.25	0.25	1	4	0.25
**6**	0.125	2	0.25	1	2	>8	0.5
**7**	0.063	4	0.125	2	0.5	>8	0.25
**6**-enant.	>8	>8	>8	>8	n.t.	n.t.	>8
Colistin	0.25	0.25	0.125	0.125	0.25	0.125	>8
Meropenem	0.063	0.063	0.063	0.063	>8	>8	0.031
CAZ-AVI	0.25	0.25	0.25	0.25	>8	>8	0.125
Gentamicin	0.5	1	0.5	0.5	>8	>8	0.5
Tigecycline	0.25	0.25	0.5	n.t.	2	n.t.	0.5

**Fig. 3.**
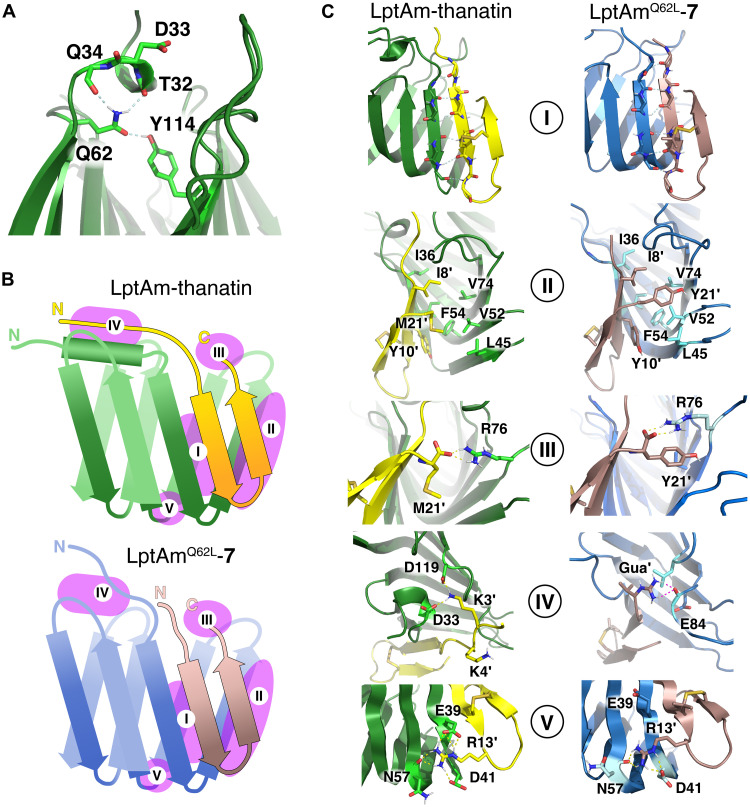
Energy-minimized average structures from MD simulations of thanatin (yellow) bound to LptAm (green) and compound 7 (brown) bound to LptAm^Q62L^ (blue). (**A**) Important interactions involving Q62 that helps to anchor the N-terminal helix to the β-jellyroll. (**B**) Schematic presentations of the fold of the LptAm-thanatin and the LptAm^Q62L^-**7** complex. Roman numbers I to V refer to the five principal sites of interaction. (**C**) Structural details of interactions at sites I to V for the LptAm-thanatin and the LptAm^Q62L^-**7** complexes. For a comprehensive comparison of all complexes described in this work, see fig. S23.

Previously, we studied a known monomeric variant of LptA (*20*), truncated at the C terminus and herein referred to as LptAm, in complex with thanatin (*16*). Refinement of the LptAm-thanatin complex nuclear magnetic resonance (NMR) structure (6GD5) with molecular dynamics (MD) simulations revealed the salt bridge interaction between the M21′ C terminus of thanatin and the R76 guanidinium group of LptA as one of the five key binding determinants of the complex (Fig. 3C, site III). In addition, electrostatic interactions of D33 and D119 with either K3′ or K4′ (fig. S26) were observed. Alanine and d–amino acid scans were conducted to identify key residues and pharmacophores, including I8′, I9′, Y10′, and R13′ (Fig. 2B). These four residues are located in the N-terminal strand of thanatin’s hairpin that docks in a parallel orientation onto the first N-terminal strand of the β-jellyroll of LptAm (Fig. 3C, site I) (*16*). The side chains of I8′, Y10′, and M21′ deeply insert into the hydrophobic core of the β-jellyroll (Fig. 3C, site II), while the R13′ guanidinium group interacts with LptAm E39, N57, and D41 (Fig. 3C, site V). Notably, analogous interactions involving the guanidinium group R159 of LptA are formed at the interface of the LptA homodimer complex (fig. S19) (*16*). Thanatin forms a β-hairpin in solution (*21*), stabilized by the C11′─C18′ disulfide bond, and linear analogs lack antimicrobial activity on *E. coli* corroborating the importance of macrocyclization (*22*).

The major mechanism of spontaneous resistance to thanatin, leading to substantial minimum inhibitory concentration (MIC) increase, involves the formation of the LptA^Q62L^ mutant (vide infra) (*16*). We hypothesized that the frequency of resistance (FOR) is lower for peptides with highest antimicrobial activity against resistant strains. Given the urgency to develop novel antibiotics with reduced resistance rates, we therefore focused on further reducing MIC values toward the thanatin-resistant clones harboring the LptA^Q62L^ mutation. Incorporation of a short–side chain cationic amino acid l-2,4-diaminobutyric acid (Dab) at positions 14′ and 19′ enhanced the antimicrobial activity against our panel of *E. coli* and *K. pneumoniae* LptA^Q62L^ mutants (Table 1). In addition, substitution of G16′ by d-Dab improved potency and in conjunction with the replacement of C11′ by l-penicillamine (Pen) further improved blood plasma stability of compound **2** (table S4A). The C-terminal residue M21′ could be replaced by the close isostere norleucine (Nle) or by an aromatic residue [WO2022028738A1, (*23*)].

Next, supported by the LptAm-thanatin complex NMR structural data (6GD5), we postulated that further truncation of the peptide N-terminal tail, K3′-P5′ (Fig. 3C, site IV), could still provide potent compounds while reducing the peptide net positive charge. Shorter peptides **3** to **7** containing 16 amino acids (Fig. 2) show comparable or improved antimicrobial activity against wild-type (WT) strains and thanatin-resistant clones compared to the 19-mer peptide **2** (Table 1). The toxicity of **3** toward mammalian cell [median inhibitory concentration (IC_50_) = 0.04 mM on HeLa cells] was substantially decreased in **6** (IC_50_ = 1.16 mM) presumably by reducing its amphiphilicity through substitution of P7′ by trans–hydroxyproline (Hyp), I9′ by Thr, and Nle21′ by Tyr (table S4A) thus mitigating potential nonspecific activity on cellular membranes. While modifying P7′ by Hyp did not negatively affect the antimicrobial activity of **4**, compound **6** containing the T9′ in place of the I9′ shows increased MIC values against the LptA^Q62L^ mutant clones. Structural studies of the complex of **5** with LptA^Q62L^ revealed I9′ as an important part of the hydrophobic interface (vide infra) contributing to binding affinity. Similar to thanatin (*16*), the all-(d)-**6** enantiomer is inactive against our panel of strains (Table 1). Last, introduction of the guanidine (Gua) moiety at the N terminus of compound **7** (Fig. 2) proved critical to achieve sufficient proteolytic stability translating into improved pharmacokinetic profile and potent in vivo efficacy (table S4, A and B).

### Structural determinants of thanatin resistance

The main cause for reduced binding of thanatin to mutant LptAm^Q62L^ involves destabilization of the N-terminal helix (V28-Q34) in LptAm. In WT-LptAm, Q62 forms hydrogen bonds to Q34, T32, and Y114 and thereby anchors the helix onto the rim of the β-jellyroll (Fig. 3A). The Q62L LptAm mutant lacks this important H-bond network, and the N-terminal helix is absent in the LptAm^Q62L^-**7** complex. This is validated by a two- to fourfold increased root mean square deviation (RMSD) fluctuations of residues V28-Q34 during MD simulations (fig. S27). In the LptAm-thanatin complex, electrostatic interactions between thanatin residues K3′ and K4′ and N-terminal helix D33 contribute to the binding. The importance of these interactions is confirmed by the fourfold decrease in binding affinity of thanatin for the engineered mutant LptAm^D33A^ [inhibition constant (*K*_i_) = 2.7 ± 0.2 nM for WT-LptAm and 10.5 ± 1.5 nM for LptAm^D33A^]. The missing interaction with the N-terminal helix accounts in part for its decrease in affinity for LptAm^Q62L^ (*K*_i_ = 2.7 ± 0.2 nM in LptAm versus 33.8 ± 5.6 nM in LptAm^Q62L^) (table S12).

The solution structure of **7** in complex with LptAm^Q62L^ explains in part the improved antimicrobial activity of **7** against the LptA^Q62L^ harboring mutant clones. The affinity of **7** for LptAm^Q62L^ is reduced compared to its affinity for WT-LptAm (*K*_i_ = 1.9 ± 0.1 nM in LptAm versus 17.2 ± 3.1 nM in LptAm^Q62L^). Nevertheless, the *K*_i_ value of **7** for LptAm^Q62L^ remains twofold lower than that of thanatin (table S12). While crucial binding determinants of the LptAm-thanatin complex are retained (Fig. 3B) in the LptAm^Q62L^-**7** complex, additional interactions are supporting the improved affinity of **7**. The introduced Y21′ forms π-π stacking interactions with Y10′ and F54, as well as hydrophobic contacts with L45 and V52 anchoring compound **7** more stably in the hydrophobic core (Fig. 3C, site II). In addition, the salt bridge between the C terminus and the guanidinium group of R76 is further stabilized by a cation-π interaction with Y21′ (Fig. 3C, site III). The loss of the N-terminal helix and the related destabilization of the binding interface does not affect binding of **7** to LptAm^Q62L^ as critically as for thanatin. Unlike thanatin, the shorter N-terminal tail of **7** does not interact with D33 and D119 of LptAm, and, therefore, the change of conformation at the N terminus of LptAm^Q62L^ has less influence on the binding strength of **7**. Accordingly, binding affinities of **7** for the engineered mutant LptAm^D33A,Q62L^ and for LptAm^Q62L^ are very similar (*K*_i_ = 17.2 ± 3.1 nM for LptAm^Q62L^ and 17.0 ± 7.3 nM for LptAm^D33A,Q62L^). Compound **7** binds to LptA^Q62L^ as the V6′-Hyp7′ cis- and trans-conformer, resulting in two sets of peaks for proximal residues (fig. S16). In the complex, the N-terminal guanidinium group according to MD simulations forms electrostatic interactions with E84 only in the cis-conformer. Together, these binding features make compound **7** a better ligand than thanatin for LptA and, in particular, for LptAm^Q62L^.

Compound **5** elicits a lower FOR compared to **7**, in line with its better activity against the LptA^Q62L^ thanatin-resistant clones. The solution structure reveals that **5** forms more contacts with the terminal β-strand of LptAm^Q62L^, particularly involving I8′ and I9′, as well as electrostatic interactions of the N-terminal amino group with E84 (fig. S23, site IV), corroborating the idea that we could reduce the FOR by optimizing the peptide binding affinity for LptA^Q62L^ (fig. S24 and table S12).

### Biological profiling

Both the lead antibiotic **7** and thanatin display a narrow activity spectrum encompassing all Enterobacteriaceae (table S5). Compared to thanatin, the compounds **4** to **7** showed markedly lower MIC values on parental and LptA^Q62L^ thanatin-resistant strains (Table 1). High primary sequence conservation of LptA within Enterobacteriaceae correlated well with the antibacterial activities of the peptides. Gram-negative bacteria with LptA sequence identity of <70% to *E. coli* were largely insensitive to thanatin and **7** (table S5), consistent with LptA as the key target.

In vitro activity against a panel of 121 Enterobacteriaceae including CRE, MDR, and XDR isolates revealed MIC_90_ values of 8, 1, and 0.5 mg/liter for thanatin, **6**, and **7**, respectively (table S6). Development of resistance against thanatin was observed after 1 day of passaging in both *E. coli* and *K. pneumoniae* and is characterized by a spontaneous FOR of 1.2 × 10^−6^ for *E. coli* at 4× MIC. Optimized analogs delayed resistance development in *E. coli* and *K. pneumoniae* to later passages, after 3 to 6 days or 3 to >10 days, respectively (fig. S8), with spontaneous frequencies of resistance at 4× MIC of 8.6 × 10^−9^, 1.3 × 10^−7^, and <3.7 × 10^−9^ in *E. coli* and 2.9 × 10^−7^, 3.8 × 10^−8^, and <3.1 × 10^−9^ in *K. pneumoniae* for compounds **6**, **7**, and **5**, respectively (table S7).

Mutation profiles of broadly selected colonies from spontaneous mutation experiments with thanatin, **4**, and **6** were determined by whole-genome sequencing and revealed that mutations within LptA were the most recurrent (table S8). Among the LptA mutations, Q62L was the prevalent mutation conferring moderate-to-high levels of resistance, while other LptA mutations appeared less frequently and generally induced smaller shifts in MIC values (table S9). While antimicrobial activity of **6** and **7** is reduced against these LptA mutants, their MIC values remain well below those of thanatin. No mutation was identified in the LPS transport bridge anchors LptC and LptD. Further mutations were observed in lipid A biosynthesis and regulation, in peptide transporters, metabolism, protein N-glycosylation, or nonannotated open reading frames (table S8) and none of them affected MIC values substantially. Most of these off-target mutations affect genes responsible for the LPS biosynthesis, suggesting that the disruption of the Lpt bridge by the thanatin analogs and subsequent accumulation of LPS at the inner membrane may account for the bactericidal effect.

### In vitro and in vivo pharmacology

All the compounds display similar plasma protein binding and are not hemolytic. The higher in vitro plasma stability of compound **7** (table S4A) translated into increased in vivo plasma exposure and improved pharmacokinetics in mice (table S4B).

Intravenous administration of **7**, twice a day (BID), was tolerated up to 30 mg/kg per day (q12h) in mice. As nephrotoxicity is often considered the main safety concern for polycationic peptides, we decided to evaluate the compounds early on in a repeat dose safety study. Compounds **6** and **7** were intravenously administered BID to mice (15 mg/kg per dose, q12h) for seven consecutive days and were well tolerated with no adverse events or detectable kidney toxicity (table S4B).

**Fig. 4.**
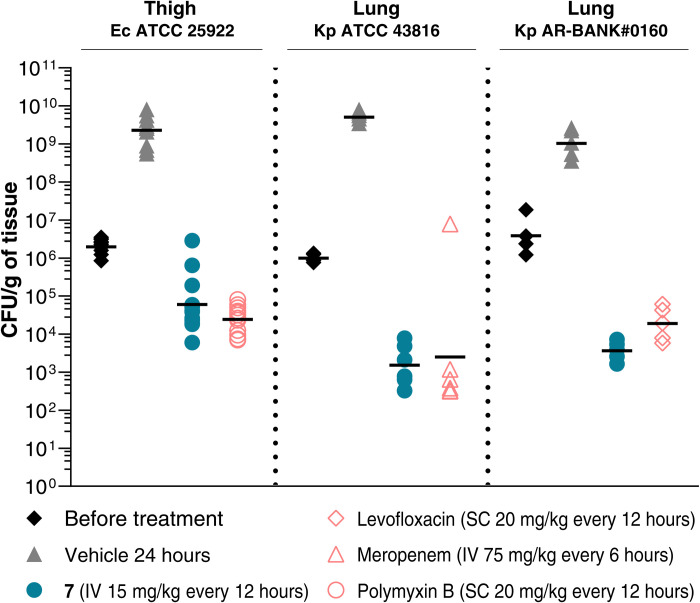
In vivo efficacy of compound 7. Compound **7** activity against *E. coli* (Ec) American Type Culture Collection (ATCC) 25922 in mouse neutropenic thigh infection model, *K. pneumoniae* (Kp) AR-BANK#0160 and *K. pneumoniae* ATCC 43816 in mouse neutropenic lung infection models. Colony-forming units (CFU) counts 24 hours after first administration of **7** (blue), vehicle only (gray), and standard of care (red) are compared. The geometric mean value of each group is depicted as a black dash.

In vitro activity translated in vivo with potent efficacy in mouse models of peritonitis (fig. S7A), thigh, and lung infections (Fig. 4). A dose response study of **7** in the *K. pneumoniae* AR-BANK#0160 lung infection model established a median effective dose of 3.4 mg/kg per day (q12h), confirming potent activity against CRE (fig. S7C). Compound **7** is more efficacious in the lung models compared to the thigh model, and additional studies are needed to explain this difference.

### Mode-of-action studies: Interactions of Lpt proteins in the periplasmic bridge

The N-terminal β-strand of LptA binds in a head-to-tail fashion to the C terminus of another LptA and to the C terminus of LptC, and the N-terminal periplasmic domain of LptD associates with the C-terminal strand of LptA (Fig. 1). To understand how thanatin analogs compete with these interactions, we determined affinities of LptA to compounds **1**, **2**, **5**, **6**, and **7** and to LptA and LptC. To unambiguously determine affinities of the LptA-LptA interaction, two monomeric LptA mutants were used, the known C-terminal truncation LptAm and a second mutant, mLptA, with three mutations at the N terminus (E39V, M47A, and R76A) (figs. S3 and S4). Using these two constructs, the *K*_i_ of the mLptA-LptAm interaction was determined by fluorescence polarization (FP) as 34.9 ± 3.0 nM.

To probe the LptA-LptC interaction, we modified a previously described periplasmic LptC construct (*24*) at two sites (Y60A and R61A) to yield a monomeric construct (*25*), LptC^AA^ (fig. S3). The well-defined heterodimer LptAm-LptC^AA^ is characterized by a *K*_i_ of 1.8 ± 0.4 μM. (fig. S5). NMR and size exclusion chromatography–multiangle light scattering (SEC-MALS) data reveal formation of the respective dimer via the suspected interface (figs. S5 and S22). The *K*_i_ values for corresponding interactions of the thanatin-resistant mutant LptAm^Q62L^ with mLptA and LptC^AA^ were 69.0 ± 14.8 nM and 26.7 ± 3.5 μM, respectively. Similar values were determined for the *K. pneumoniae* proteins (table S11).

### Disassembly of the periplasmic bridge by compound 7

**Fig. 5.**
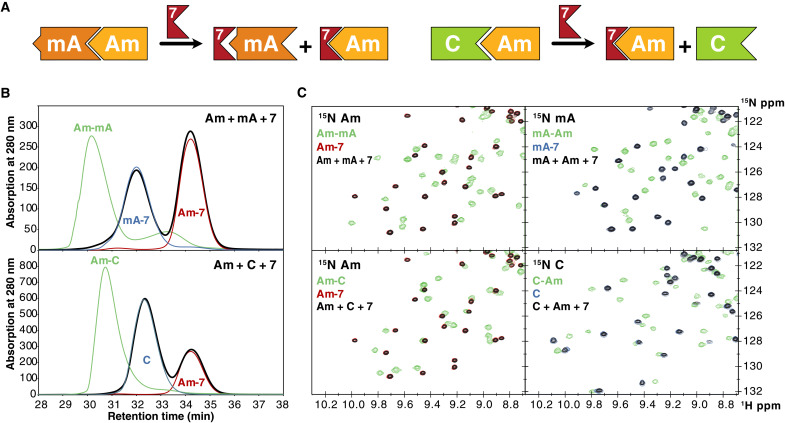
Disassembly of *E. coli* dimers. (**A**) Schematic overview of the disassembly process. A, LptA; C, LptC^AA^. (**B**) SEC trace from the mLptA-LptAm (top) and LptAm-LptC^AA^ (bottom) mixtures in presence of compound **7** (bold black lines). SEC traces of the protein dimers (green lines) or from the **7** complexes with LptAm, mLptA, or LptC^AA^ are shown (thin blue and red lines). (**C**) [^15^N,^1^H]-HSQC spectra from the mLptA-LptAm (top) and LptAm-LptC^AA^ (bottom) mixtures in presence of **7**. In presence of **7**, the peaks of mixtures of the dimeric proteins superimpose with those of the corresponding monomeric proteins. In both NMR and SEC experiments, the protein concentration was 200 μM, and 1.5 equivalent of peptide were added. For a complete set of all SEC or NMR data, see fig. S21.

Next, we used SEC and NMR to probe the integrity of protein-protein interactions upon addition of compound **7**. The SEC chromatogram reveals that peaks from the dimeric species disappear and two new peaks from the monomers arise when **7** is added to the protein mixture (Fig. 5B). We complemented the SEC studies by [^15^N,^1^H]–heteronuclear single-quantum coherence (HSQC) NMR experiments. Two sets of experiments were measured for each protein-protein pair, in which one of the two interacting proteins was uniformly ^15^N labeled, while the cognate partner was unlabeled and vice versa. Representative NMR spectra and SEC traces in Fig. 5C demonstrate that both the LptAm-mLptA and the LptAm-LptC^AA^ interactions were completely disrupted by **7** in agreement with previous reports on thanatin (*17*). Upon addition of **7**, signals from LptAm-mLptA and LptAm-LptC^AA^ perfectly superimpose with those from LptAm-**7**, mLptA-**7**, or LptC^AA^.

The *K*_i_ for the LptAm-**7** interaction of 1.9 ± 0.1 nM is comparable to the *K*_i_ of the LptAm-thanatin complex of 2.7 ± 0.2 nM and is 18 times stronger than the LptAm-mLptA interaction (*K*_i_ = 34.9 ± 3.0 nM). In contrast, thanatin binds to LptAm^Q62L^ with a *K*_i_ of 33.8 ± 5.6 nM that is only two times stronger than the mLptA-LptAm^Q62L^ interaction (*K*_i_ = 69.0 ± 14.7 nM). Compound **7** binds LptAm^Q62L^ with a higher affinity of 17.2 ± 3.1 nM and hence four times stronger than the mLptA-LptAm^Q62L^ interaction. In agreement, SEC chromatograms reveal that thanatin fails to completely disassemble the mLptA-LptAm^Q62L^ complex, whereas **7** achieves near complete disassembly (fig. S21).

## DISCUSSION

A comprehensive medicinal chemistry effort around the thanatin molecular scaffold, combining classical phenotypic with structure-guided optimization, resulted in a promising lead series that pioneer a novel class of Gram-negative antibiotics. The lead compound **7** is bactericidal against MDR and XDR Enterobacteriaceae including carbapenemase- and colistin-resistant strains and, hence, potentially addresses critical limitations observed in the antimicrobial spectrum of current SoC antibiotics in the clinic. The potent in vitro activity and optimized drug-like ADME and pharmacokinetic (PK) properties of **7** translate into potent antimicrobial in vivo activity in various mouse infection models. Detailed mechanistic studies established the competitive disruption of LptA-LptA and LptA-LptC interactions as the mechanism of action for the thanatin analogs. This work further validates the bacterial Lpt machinery and, more specifically, its periplasmic components, as a promising and druggable target for the development of novel antibiotics.

Resistance to clinically used antibiotics is often related to off-target effects such as up-regulation of efflux pumps or in vivo drug modifications (*26*). In contrast, whole-genome sequencing of a diverse panel of thanatin-resistant strains confirmed on-target modifications on LptA as the main resistance determinant in *E. coli* and *K. pneumoniae*. Among the LptA mutations, the Q62L is the most frequent mutation that confers a significant level of resistance to thanatin. The NMR structure of the LptAm^Q62L^-**7** complex reveals that the Q62L mutation induces a conformational change in the N-terminal part of LptA resulting in the disruption of contacts to thanatin, thereby reducing its affinity for LptA and, hence, its antimicrobial activity. Thanatin’s large molecular footprint allowed us to modify the peptide sequence to limit this loss of affinity by strengthening the interaction between the compound and LptA at the primary binding site at the LptA-LptA/LptC interface. Concomitantly, the residues nonessential for binding were modified to achieve favorable safety and pharmacokinetic properties. Structure-based molecular design, combined with screening against a panel of LptA^Q62L^ clones helped us to identify compounds **5**, **6**, and **7** with increased antimicrobial activity and lower FOR (range from 10^−7^ to 10^−9^ for **5**, **6**, and **7** versus 10^−6^ for thanatin at 4× MIC). The improved antimicrobial activity against the LptA^Q62L^ mutants generally correlates with tighter binding to LptAm^Q62L^. However, there were still exceptions, indicating that additional factors such as membrane permeation, periplasmic stability, or binding to LptD (*19*) were most likely also optimized and will be subject of future investigations.

The antibacterial activity spectrum of our macrocyclic peptides is related to the sequence identity with *E. coli* LptA. This suggests that pathogen-specific modifications of the peptide sequence to target LptA of other important Gram-negative bacteria may be achievable. The attractive in vitro and in vivo profile of these new antibiotics, coupled with their novel mechanism of action, showing no cross-resistance to standard of care antibiotics, may provide clinicians with additional treatment options to fight AMR, either in combination with SoC or as stand-alone antibiotics.

## MATERIALS AND METHODS

### Design and cloning of the protein constructs

All constructs were cloned into a pEM3BT2 vector. Polymerase chain reaction (PCR) was performed on the genomic extract (obtained with the genomic DNA mini kit from Invitrogen) of *E. coli* American Type Culture Collection (ATCC) 25922 and *K. pneumoniae* ATCC 13883. The vector and the PCR products were digested with Sap I and Bam HI (New England Biolabs), purified in a 1% agarose gel, and ligated with T4 DNA Ligase (Fermentas). Point mutations were introduced into the vector constructs via PCR with primers containing the point mutation. All primers are summarized in table S1.

To increase stability and solubility, all proteins were expressed as C-terminal fusions to the highly soluble 62-residue protein G (GB1) from *Streptococcus* sp. (*27*). Proteins of interest were cleaved from GB1 by Tobacco etch virus (TEV) protease leaving only an N-terminal glycine as an additional amino acid. For exact sequences, see table S2.

The monomeric N-terminal truncated LptA version (LptAm) was previously used in Vetterli *et al.* (*16*). The resistant mutant LptAm^Q62L^ contained only the Q62L mutation when compared to LptAm. The mutant mLptA was designed to avoid formation of dimer contacts with LptA at its N terminus. During the design, a model of the dimer was made, based on Protein Data Bank (PDB) entry 2R19, and an alanine screen was performed using the program package MOE to obtain residue-specific ΔStability and ΔAffinity values. The best hits (high-affinity change and low-stability change) were expressed, and the LptA_25–185_ mutant E39V M47A R76A was selected as the best candidate. LptC Y60A R61A (LptC^AA^) is composed of only the periplasmic domain of LptC_25–191_ (truncated after the transmembrane helix). The previously described double mutation Y60A and R61A were introduced to avoid head-to-head dimerization (fig. S3) (*25*).

All mutations and truncations of the *E. coli* constructs could be transferred to the *K. pneumoniae* sequences due to their high sequence homology. The corresponding *K. pneumoniae* proteins were purified with similar yields and displayed similar properties.

### Expression and purification

*E. coli* BL21 (DE3) cells were transformed with the respective plasmid, and the protein constructs were expressed in M9 medium prepared as described in Schuster *et al.* (*28*). Usually, ^15^NH_4_Cl (1.2 g/liter) and glucose (5 g/liter) were used for ^15^N labeling, whereas ^15^NH_4_Cl (1 g/liter) and U-^13^C-glucose (3 g/liter) were used for ^13^C,^15^N labeling. The cells were induced with 0.5 mM isopropyl-β-d-thiogalactopyranoside at an optical density at 600 nm of 0.6 to 1.0 and incubated overnight at 25°C. Cells were harvested by centrifugation at 5000*g*, flash-frozen in liquid nitrogen, and stored at 80°C until further use.

All steps of the purification were performed on ice or at 4°C. Cells were resuspended in 5 ml/g by vortexing in resuspension buffer [20 mM Na-phosphate (pH 8), 300 mM NaCl, 10% glycerol, 5 mM MgCl_2_, deoxyribonuclease (0.05 mg/ml), and lysozyme (3 mg/ml)]. Afterward, the same amount of lysis buffer [80 mM Na-Pi (pH 8), 300 mM NaCl, 10% glycerol, 2% Triton X-100, 2% Na-cholate, 40 mM imidazole, and 1 mM phenylmethylsulfonyl fluoride] was added, and incubation was continued for 2 hours. The lysate was cleared by centrifugation for 30 min at 40000*g*, and the supernatant was loaded on 1 ml of Ni–nitrilotriacetic acid (NTA) superflow resin per 10 ml of lysate. The resin was washed with 15 column volumes (CVs) of wash buffer [20 mM Na-Pi (pH 8), 300 mM NaCl, 10% glycerol, 30 mM imidazole, and 20 mM Na-cholate] and 15 CVs of Lpt buffer [20 mM Na-Pi (pH 8), 300 mM NaCl, and 10% glycerol]. The protein was eluted with 5 CVs of elution buffer [20 mM Na-Pi (pH 7.5), 300 mM NaCl, and 300 mM imidazole]. To remove the His_6_-GB1 tag, 1 mg of TEV protease was added to 10 ml of elution together with 1 mM EDTA and one-eighth of a cOmplete Mini protease inhibitor tablet. The solution was then dialyzed overnight against SEC buffer [20 mM Na-Pi (pH 7) and 150 mM NaCl] in a dialysis membrane with a 6- to 8-kDa cutoff. The Ni-NTA superflow resin was equilibrated with SEC buffer, and the flow-through was loaded two times to bind the His-tagged GB1 and TEV protease as well as impurities. After washing with 1 CV of SEC buffer, the flow-through was concentrated in an Amicon Ultra 15 with a 10-kDa cutoff filter. The protein concentration was determined using a Nanodrop. The yields of the expressed Lpt proteins in M9 expression medium were 15 to 70 mg/liter. Purified proteins were characterized by SDS–polyacrylamide gel electrophoresis (fig. S1), mass spectrometry (MS) (fig. S2), and SEC-MALS (figs. S4 and S5). The final concentration for the NMR samples was 200 to 600 μM in SEC buffer.

### Synthesis of thanatin and derivatives 1 to 7

Peptides were synthesized by solid-phase peptide synthesis (SPPS) based on a mixed solid/solution phase procedure using the Fmoc/tBu strategy. Fmoc-Tyr(tBu)-OH or Fmoc-Nle-OH or Fmoc-D-Tyr(tBu)-OH was loaded onto 2-chlorotritylchloride resin in dichloromethane in presence of *N*,*N*-diisopropylethylamine. After capping [methanol (MeOH)], a typical loading of 0.4 to 0.8 mmol/g was achieved. The peptide was assembled by standard automated Fmoc SPPS on 0.025-mmol scale (based on preloaded resin loading). Fmoc groups were deprotected with 20% piperidine in dimethylformamide (DMF). Amino acids were introduced by double coupling of 2× 15 min (2× 30 min after synthesis cycle 9) with amino acid/2-(7-aza-1*H*-benzotriazole-1-yl)-1,1,3,3-tetramethyluronium hexafluorophosphate (HATU)/*N*-methylmorpholine (NMM) of 8/7.6/24 molar equivalents (eq), respectively, in *N*-methyl-2-pyrrolidone (NMP). For the unusual unnatural amino acid derivatives, e.g., Fmoc-Lys(azido-PEG4)-OH, the coupling was performed only once for 2 hours with amino acid/HATU/NMM of 4/3.8/12 eq in NMP. Each coupling step was followed by a capping step with acetic anhydride (Ac_2_O)/NMM of 12/12 eq in NMP. The synthesis cycle Fmoc deprotection/coupling/capping was repeated with the appropriate amino acid until the end of the elongation.

To introduce the N-terminal guanidine group (Gua) in **7**, *N*,*N*′-bis-Boc-1-guanylpyrazole (10 eq) dissolved in a mixture of 0.5 ml of dimethylsulfoxide (DMSO)/DMF of 1/1 (v/v) was added to the peptide resin after removal of the Fmoc of the last coupled amino acid (V6′; see Table 1). The resin suspension was shaken overnight at room temperature and washed with DMF.

After final Fmoc removal of the last coupled amino acid (V6′) or N terminus functionalization (Gua), the resin was washed with dichloromethane. The peptide was simultaneously cleaved from the resin and globally deprotected (removal of side chain protecting groups) by treatment with 6 ml of trifluoroacetic acid (TFA)/triisopropylsilane/thioanisole/anisole/water of 82.5/5/5/5/2.5 (v/v/v/v/v). After 2.5 hours, the resin was filtered, and the crude linear peptide was precipitated in 35 ml of cold diethyl ether (Et_2_O). The solid was isolated by centrifugation and washed three times with 20 ml of Et_2_O. After removal of residual Et_2_O by evaporation, the crude cyclic product was dissolved in 1.8 ml of water and 1.8 ml of ammonium acetate buffer (1 M, pH 6) containing 4% of DMSO (v/v). The disulfide bridge formation was monitored by liquid chromatography (LC)–MS and Ellman’s colorimetric test. Reaction time required to reach completion varied from 2 to 6 days depending on the peptide sequence. The crude cyclic product was purified by preparative reversed-phase liquid chromatography (RP-HPLC) (0.1% TFA in water/acetonitrile gradient, C18 column material). For analytical data, see table S3.

### Synthesis of fluorescently labeled compounds 6-FL

The peptide precursor bearing the **6**-azido-l-lysine derivative (fig. S6) was synthesized and purified as described above. The purified peptide (10 mg, TFA salt) in water (0.7 ml) was submitted to a copper-catalyzed azide-alkyne cycloaddition with 1 eq of the Alexa Fluor 647 alkyne (Fluoroprobes) in degassed MeOH (20 mg/ml) and a mixture of 1 eq of copper (II) sulfate pentahydrate (CuSO_4_*5H_2_O) (11 mg/ml) and 3 eq ascorbic acid (23 mg/ml) in water. The reaction concentration was close to 10 mg/ml of peptide. After 1 hour, partial precipitation of the Alexa Fluor 647 alkyne was observed. Additional degassed MeOH (0.5 ml) was added, and the reaction was stirred overnight. MeOH was removed by evaporation under reduced vacuum, and the crude peptide was purified by RP-HPLC (0.1% TFA in water/acetonitrile gradient, C18 column material). For analytical data, see table S3.

### Biological profiling of the compounds

To access the pharmacological profile, the hemolytic behavior, cytotoxicity, plasma stability, and plasma protein binding were measured. All data were determined in biological replicates.

To test their hemolytic potential, compounds were incubated in the presence of phosphate-buffered saline (PBS)–washed human red blood cells. After 1-hour incubation at 200 mg/liter and 37°C, the samples were centrifuged (3220*g*), the supernatants were diluted in Dulbecco’s PBS, and optical density at 540 nm was determined. The hemolysis induced by the compound was calculated versus a 100% lysis control prepared with 2.5% Triton X-100 (table S4A).

The cytotoxicity of compounds was determined by determining the number of viable cells using the Sigma Cell Counting Kit-8 (WST-8). HeLa cells were incubated at 37°C and 5% CO_2_ for 24 hours, after which the medium was replaced with fresh medium containing dilutions of compounds. After another 48 hours, cell viability was monitored by addition of WST-8 solution and measurement of optical density at 450 nm (table S4A).

To determine compound stability in plasma, compounds were incubated for 0, 15, 30, 60, 120, and 240 min in K_3_EDTA-stabilized plasma of human or CD-1 mice (BioIVT). Samples were extracted by precipitation with 3 volumes of acetonitrile + 0.5% TFA, and compound stability was determined by relating residual with initial compound amount quantified by LC–MS/MS (table S4A).

Plasma protein binding was determined by the mass balance method using a 30-kDa cutoff filter to separate free from plasma protein–bound compounds in pooled human and mouse plasma samples. The compounds were diluted in pH 7.5–adjusted plasma to a final concentration of 10 mg/liter and incubated for 30 min at 37°C. After ultrafiltration, protein binding was determined by subtracting the percentage of compound in ultrafiltrate from the total amount of compound in spiked plasma (table S4A).

### Tolerability and 7-day repeated dosing in mice

Two different study designs were used to determine the potential toxicity of these compounds, when given BID via intravenous bolus (injection) 12 hours apart to CD-1 mice. In the first type of study, male mice were dosed on a single day at three escalating dose levels (three mice per group), while the second type of study male mice (nine mice per group) were dosed for seven consecutive days with the same administration regimen (q12h). Mice were observed for mortality, cage side observations, individual body weights, and food and water consumption from their arrival to the facility to the necropsy. Detailed clinical observations were conducted up to at least 3 hours after the first daily dosing and up to at least 1 hour after the second daily dosing. In addition, the Irwin battery of tests was used to assess the mice neurobiological and physiological state during the single day study at 30, 60, 120, 180, and 240 min after the first administration. The following parameters were included in the observations: occurrence of abnormal gait, catalepsy, piloerection, abnormal respiration, increase or decrease in spontaneous activity, spontaneous activity following audible stimuli (tapping on side of cage), touch response, and body tonus. Any additional symptoms observed were also reported. Symptoms were scored from 0 to 3, where 0 represents no findings and 3 the highest score, and frequencies of animals exhibiting symptoms were recorded (table S4C).

### In vivo pharmacokinetic study

PK were studied in adult CD-1 male mice following either a single dose of subcutaneous (sc) injection (10 mg/kg) or by a single dose of intravenous (iv) (5 mg/kg; bolus) injection to the tail vein. Plasma samples were taken from nine mice in each treatment group at 0.25, 0.5, 1, 2, 3, 4, and 8 hours after dose (for single subcutaneous administration) or at 0.083, 1, 2, 4, 8, and 12 hours after dose (for single intravenous administration). Blood samples (in Li-heparin as anticoagulant) were collected from the retrobulbar venous plexus under short isoflurane anesthesia. Plasma samples were obtained by centrifugation for 10 min at 3000*g* and 4°C, and the supernatant was analyzed by LC MS/MS. After separation on a phenyl-hexyl reversed-phase column using an acetonitrile-water gradient, peaks were analyzed by electrospray ionization–MS. The mean plasma concentration and the SD from all three mice within each time point were calculated, and PK parameters of test agent were estimated using a noncompartmental analysis model with trapezoid area calculation (table S4B).

### Mouse models of peritonitis, lung, and thigh infections

To evaluate the in vivo efficacy of compound **7** to treat peritoneal infections, adult immunocompetent female ICR mice (eight per group) were infected on day 0 by intraperitoneal (ip) administration of *E. coli* ATCC 25922 [1.54 × 10^5^ colony-forming units (CFU)/ml] with 5% mucin into the peritoneum. The vehicle saline was intravenously administered BID at 1 and 13 hours after infection (q12h). Compound **7** was intravenously administered BID at 1 and 13 hours after infection (q12h) at 15 and 30 mg/kg per day. Control reference agent, levofloxacin at 1 mg/kg was subcutaneously administered once a day at 1 hour after infection. The animals were monitored for survival up to 7 days, BID. The significance of the survival rate was assessed with the Fisher’s exact test (fig. S7A).

To evaluate the in vivo efficacy of compound **7** to treat thigh infections, male CD-1 mice (six per group) were rendered neutropenic with intraperitoneal injections with cyclophosphamide on day −4 (150 mg/kg) and day −1 (100 mg/kg). Mice were infected 24 hours after the second dose of immunosuppressive agent by intramuscular instillation with *E. coli* ATCC 25922 diluted to an optimal concentration with PBS. For infection, mice were temporarily anesthetized using inhaled isoflurane (2.5% isoflurane/97.5% oxygen). Anesthetized mice were infected with 0.05 ml of inoculum by intramuscular instillation into each lateral thigh muscle. The inoculum concentration was 3.03 × 10^6^ CFU/ml (1.52 × 10^5^ CFU per thigh). Compound **7** was intravenously administered at a total dose level of 30 mg/kg per day of fractionated q4h, q6h, and q12h equating to individual doses of 5, 7.5, and 15 mg/kg, respectively. Control reference agent, polymyxin B, was subcutaneously administered at a total dose level of 40 mg/kg per day, at 2 and 14 hours after infection (q12h). Additional groups were included that were euthanized before treatment (2 hours after infection) or treated with vehicle [intravenous 0.9% saline BID at 2 and 14 hours after infection (q12h)] only. At 26 hours after infection, the clinical condition of all mice was assessed, the mice were euthanized by pentobarbitone overdose, and thigh tissue was harvested, weighed, and homogenized. Thigh-sample homogenates were quantitatively cultured onto agar for determination of the counts of CFU per gram of thigh (fig. S7B). Data from the efficacy study were analyzed using StatsDirect software (version 3.1.8). The nonparametric Kruskal-Wallis test was used to test all pairwise comparisons (Conover-Inman) for tissue burden data. For the purposes of this study, each thigh was considered an independent infection site, generating two data points per mouse.

To evaluate the in vivo efficacy of compound **7** to treat lung infections against *K. pneumoniae* AR-BANK #0160, female ICR mice (five per group) were rendered neutropenic with intraperitoneal injections with cyclophosphamide on day −4 (150 mg/kg) and day −1 (100 mg/kg). Mice were infected 24 hours after the second dose of immunosuppressive agent by intranasal injection of *K. pneumoniae* AR-BANK #0160 inocula with a total volume of 20 μl, 10 μl per nostril (1.02 × 10^6^ CFU/ml). Compound **7** was intravenously administered BID, at 2 and 14 hours after infection (q12h), at 0.5, 1, 2, 4, 8, 15, and 30 mg/kg per day. Control reference agent, levofloxacin, at 40 mg/kg per day was subcutaneously administered BID, at 2 and 14 hours after infection (q12h). Additional groups were included that were euthanized before treatment (2 hours after infection) or treated with vehicle [intravenous 0.9% saline BID at 2 and 14 hours after infection (q12h)] only. At 26 hours after infection, the clinical condition of all mice was assessed, the mice were euthanized by CO_2_ asphyxiation, and lung tissue was harvested, weighed, and homogenized. Pathogen burden was enumerated with the serial dilution plating technique. The CFU value per grams of tissue was calculated and compared to a vehicle group (fig. S7C). Significance of effects was assessed with one-way analysis of variance (ANOVA), followed by Dunnett’s test.

To evaluate the in vivo efficacy of **7** to treat lung infections against *K. pneumoniae* ATCC 43816, male ICR mice (six per group) were rendered neutropenic with injections with cyclophosphamide on day −4 (200 mg/kg) and day −1 (150 mg/kg). Mice were infected 24 hours after the second dose of immunosuppressive agent by intranasal injection of *K. pneumoniae* ATCC 43816. For infection animals were anesthetized with a ketamine (50 mg/kg) and medetomidine (0.5 mg/kg) anesthetic cocktail via intraperitoneal injection (10 ml/kg). Anesthetized mice were infected with 0.04 ml of inoculum by intranasal instillation into mouse nostrils (20 μl per nostril, 5 min between nostrils). The inoculum concentration was 1.97 × 10^6^ CFU/ml (7.87 × 10^4^ CFU per mouse). Once the procedure had been completed, the anesthetic reversal agent atipamezole was subcutaneously administered at 3.75 mg/kg. Compound **7** was intravenously administered BID, at 2 and 14 hours after infection (q12h) at 0.5, 1, 2, 4, 8, 15, and 30 mg/kg per day. Control reference agent, meropenem, at 300 mg/kg/day was intravenously administered four times a day q6h, at 2, 8, 14, and 20 hours after infection. Additional groups were included that were euthanized before treatment (2 hours after infection) or treated with vehicle [intravenous 0.9% saline BID at 2 and 14 hours after infection (q12h)] only. At 26 hours after infection, the clinical condition of all mice was assessed, the mice were euthanized by pentobarbitone overdose, and lung tissue was harvested, weighed, and homogenized. Pathogen burden was enumerated with the serial dilution plating technique. The CFU value per gram of tissue was calculated and compared to a vehicle group (fig. S7D). Data from the efficacy study were analyzed using StatsDirect software (version 3.1.8). The nonparametric Kruskal-Wallis test was used to test all pairwise comparisons (Conover-Inman) for tissue burden data.

### Microbiology

MIC assays [Clinical and Laboratory Standards Institute (CLSI) methodology] and serial passage studies were performed as described by Luther *et al.* (*6*). The spontaneous mutation frequencies of thanatin and analogs using reference strains of *E. coli* (ATCC 25922) and *K. pneumoniae* (ATCC 43816) were determined by inoculating agarose containing the compounds at concentrations 4-, 8-, and 16-fold over the MIC. Resistant mutants were isolated, and their MIC values were determined and sent to Microbes NG (Birmingham, United Kingdom) for whole-genome sequencing and bioinformatic analysis.

### Binding assays by FP

All binding affinity assays were performed in triplicates and executed at ambient temperature (25°C) on Optiplate-96F microplates (PerkinElmer). Protein stocks were purified and concentrated in buffer 1 [50 mM Na-Pi (pH 7) and 150 mM NaCl] and, if necessary, diluted to desired concentrations using buffer 2 [50 mM Na-Pi (pH 7), 150 mM NaCl, and 0.05% Tween 20]. Peptide stocks and fluorescently labeled peptide stocks were made in buffer 2. Every serial dilution consisted of 24 wells, the first well with the highest concentrate and the last well (well 24) with the lowest. First, 50 μl of buffer 2 was added to the wells 2 to 24 of each serial dilution. Then, 100 μl of concentrated unlabeled peptide/protein in their respective buffers was added to the first well. Fifty microliters were taken from the first well and added to the second well for a 1:1 serial dilution and continued for the 24 wells. Concentrations of the unlabeled peptide/protein competitor in the first well ranged from 150 μM to 2 mM. Subsequently, 50 μl of 2× stock of the fluorescently labeled **6**-FL for the direct assays or 50 μl of 2× stock of the 1:1 mixture of the Lpt protein and **6**-FL for the indirect assays were added to each of the 24 wells. For the direct binding assays, 2× concentration stocks of **6**-FL were 2 nM for LptAm and 70 nM for LptAm^Q62L^ measurements. For indirect binding assays, 2× stock for all 1:1 protein and **6**-FL mixtures were 70 nM. Plates were covered with adhesive seals and left to incubate overnight at 4°C before FP measurements the next day. Anisotropy was measured using the Tecan Safire2 plate reader using a 10-nm bandwidth and setting the excitation and emission wavelengths to 635 and 670 nm, respectively. The G-factor (*G*) was also determined empirically as 1.094 for the instrument and included in all the anisotropy calculations.

For direct binding assays, fluorescence anisotropy (*r*) was calculated using the parallel and perpendicular polarized intensities (*I*) by the equation

$$r=\frac{I_{∥}-I_{⊥}*G}{I_{∥}+2I_{⊥}*G}$$

Anisotropy for the blank, the fluorescently labeled peptide only (**6**-FL only), was measured and subtracted from *r* to yield *r*′. The *r*′ data were normalized and fit to the One site − Total model on GraphPad Prism 9. A nonspecific binding factor (NS) was determined using a linear regression model (*y* = NS ∗ *X* + *b*) at the highest concentrations and incorporated into the fit. The dissociation constants (*K*_d_) were calculated using the total protein concentrations using the equation

$$y=\frac{B_{\max}*X}{K_{d}+X}$$

For the indirect binding assays, *r* was calculated in the same manner as the direct binding assays. To control for nonspecific binding between the fluorescently labeled peptide (**6**-FL) and competitors, **6**-FL alone was added to a replicate dilution series of the unlabeled peptide on the same plate and in triplicates with the unlabeled protein competitors on a separate plate. The control was subtracted from *r* to yield *r*′. Data were fit to a sigmoidal interpolation model on GraphPad Prism 9, and an IC_50_ was generated for each unlabeled peptide or protein measured. The *K*_i_ values were calculated using the Cheng-Prusoff equation

$$K_{i}=IC_{50}\;*{\left(1+\frac{L_{T}}{K_{d}}\right)}^{-1}$$

in which the total concentration of **6**-FL (*L*_*T*_) was used and the *K*_d_ previously derived from the peptide’s cognate direct binding assay.

### NMR spectroscopy, assignments, and structure calculations

Backbone chemical shifts of **7**-bound *E. coli* LptAm, LptAm^Q62L^, *K. pneumoniae* LptAm, and free LptC^AA^ were assigned using spectra obtained from a standard set of three-dimensional (3D) triple-resonance experiments using ^13^C,^15^N-labeled proteins (*29*). The protein-free peptide **7** was dissolved in acetate buffer, pH 4.0, and assigned using standard 2D homonuclear NOESY, COSY, and TOCSY spectra. The structure of the free peptide was calculated from the 300-ms NOESY spectrum. To unambiguously distinguish the intermolecular from the intramolecular NOEs, differently labeled LptAm and thanatin were used in Vetterli *et al.* (*16*). In the case of **7**, this was not possible because the peptide contains unnatural amino acids. Therefore, ^2^H,^15^N-LptAm constructs were grown in D_2_O using perdeuterated glucose, in which the residual proton density was less than 1%. This sample in combination with **7** allowed us to record 2D [^1^H,^1^H], ^15^N-filtered, NOESY spectra (*30*) and to obtain intraligand NOEs exclusively. Intermolecular side-chain NOEs were extracted from a ^13^C-edited, ^13^C filtered NOESY experiment. Upper-distance restraints were derived from 80-ms ^15^N and two sets of ^13^C-resolved 3D NOESY spectra, centered on aliphatic [39 parts per million (ppm)] and aromatic (120 ppm) carbons. NOESY spectra were iteratively automatically assigned using the CYANA macro noeassign (*31*). Additional torsion angle restraints were derived from backbone chemical shifts using the program TALOS+ (*32*). For more details of the structure calculations, see table S13.

### MD simulation

MD simulations were conducted using the program package Gromacs 2020 (*33*), that uses the CHARMM36-jul2020 force field (*34*). Parameters for the unnatural amino acids (Gua-Val, Hyp, Dab, and Pen) were inferred from the existing CHARMM force field parameters. The NMR-derived starting structures were first minimized by steepest descent. Thereafter, each model was placed in a periodic dodecahedron water box with a distance between the protein complex and periodic box edges of 1.0 nm. Each model was solvated with simple point charge (SPC) water, and the net charge of the system was adjusted to zero by adding counter ions (Na^+^ or Cl^−^). The cutoff radius for calculation of the van der Waals interactions was 1.0 nm, and neighbor lists within a radius of 1.2 nm were updated every 20 fs. Long-range electrostatic interactions were treated with a Particle-Mesh Ewald summation. The radius for calculating short-range electrostatic interactions was 1.2 nm. After initial minimization of the entire system, a short dynamics run at constant volume and temperature (300 K) was conducted for 100 ps using a 2-fs time step. Positional restraints with force constants of 1 × 10^5^ kJ/mol per square nanometer were initially applied to all protein atoms during the equilibration phase. Bond lengths were restrained with the LINCS algorithm using one iteration and four order expansions. The second (equilibration) dynamics run was done at constant temperature and pressure (NPT) for another 100 ps to adjust the size of the periodic cell. MD production runs had total lengths of 2 μs and integration steps of 2 fs. In all dynamics steps, the temperature of the protein complex and solvent was maintained separately at 300 K by coupling with Berendsen thermostat. The coupling time was 0.1 ps. In all NPT simulations, including the production run, the pressure was maintained at 1 atm with isotropic pressure coupling using the Parinello-Rahman algorithm, a time constant of 2 ps and a compressibility of 4.5 × 10^−5^ bar. Trajectories were analyzed using the GROMACS trajectory analysis utilities.

To improve electrostatic interactions, MD simulations were performed. Starting from the lowest-energy NMR conformer, 2-μs trajectories were computed and clustered. A suitable 200-ns cluster was then extracted that displays a low RMSD to the starting structure and occurs frequently during the simulation. Coordinates were averaged, energy-minimized, and chosen to visualize characteristic three-dimensional structure details of protein-ligand complexes in Fig. 3 and fig. S23 using the program PyMOL.

## Notes

View/request a protocol for this paper from *Bio-protocol*.

## References

[1] Antimicrobial Resistance Collaborators, Global burden of bacterial antimicrobial resistance in 2019: A systematic analysis. Lancet 399, 629–655 (2022). 35065702 10.1016/S0140-6736(21)02724-0 PMC8841637

[2] H. W. Boucher, G. H. Talbot, J. S. Bradley, J. E. Edwards, D. Gilbert, L. B. Rice, M. Scheld, B. Spellberg, J. Bartlett, Bad bugs, no drugs: No ESKAPE! An update from the Infectious Diseases Society of America. Clin. Infect. Dis. 48, 1–12 (2009). 19035777 10.1086/595011

[3] E. Tacconelli, E. Carrara, A. Savoldi, S. Harbarth, M. Mendelson, D. L. Monnet, C. Pulcini, G. Kahlmeter, J. Kluytmans, Y. Carmeli, M. Ouellette, K. Outterson, J. Patel, M. Cavaleri, E. M. Cox, C. R. Houchens, M. L. Grayson, P. Hansen, N. Singh, U. Theuretzbacher, N. Magrini, A. O. Aboderin, S. S. al-Abri, N. Awang Jalil, N. Benzonana, S. Bhattacharya, A. J. Brink, F. R. Burkert, O. Cars, G. Cornaglia, O. J. Dyar, A. W. Friedrich, A. C. Gales, S. Gandra, C. G. Giske, D. A. Goff, H. Goossens, T. Gottlieb, M. Guzman Blanco, W. Hryniewicz, D. Kattula, T. Jinks, S. S. Kanj, L. Kerr, M. P. Kieny, Y. S. Kim, R. S. Kozlov, J. Labarca, R. Laxminarayan, K. Leder, L. Leibovici, G. Levy-Hara, J. Littman, S. Malhotra-Kumar, V. Manchanda, L. Moja, B. Ndoye, A. Pan, D. L. Paterson, M. Paul, H. Qiu, P. Ramon-Pardo, J. Rodríguez-Baño, M. Sanguinetti, S. Sengupta, M. Sharland, M. Si-Mehand, L. L. Silver, W. Song, M. Steinbakk, J. Thomsen, G. E. Thwaites, J. W. M. van der Meer, N. van Kinh, S. Vega, M. V. Villegas, A. Wechsler-Fördös, H. F. L. Wertheim, E. Wesangula, N. Woodford, F. O. Yilmaz, A. Zorzet, Discovery, research, and development of new antibiotics: The WHO priority list of antibiotic-resistant bacteria and tuberculosis. Lancet Infect. Dis. 18, 318–327 (2018). 29276051 10.1016/S1473-3099(17)30753-3

[4] L. S. Tzouvelekis, A. Markogiannakis, E. Piperaki, M. Souli, G. L. Daikos, Treating infections caused by carbapenemase-producing Enterobacteriaceae. Clin. Microbiol. Infect. 20, 862–872 (2014). 24890393 10.1111/1469-0691.12697

[5] N. Srinivas, P. Jetter, B. J. Ueberbacher, M. Werneburg, K. Zerbe, J. Steinmann, B. van der Meijden, F. Bernardini, A. Lederer, R. L. A. Dias, P. E. Misson, H. Henze, J. Zumbrunn, F. O. Gombert, D. Obrecht, P. Hunziker, S. Schauer, U. Ziegler, A. Käch, L. Eberl, K. Riedel, S. J. DeMarco, J. A. Robinson, Peptidomimetic antibiotics target outer-membrane biogenesis in *Pseudomonas aeruginosa*. Science 327, 1010–1013 (2010). 20167788 10.1126/science.1182749

[6] A. Luther, M. Urfer, M. Zahn, M. Müller, S. Y. Wang, M. Mondal, A. Vitale, J. B. Hartmann, T. Sharpe, F. L. Monte, H. Kocherla, E. Cline, G. Pessi, P. Rath, S. M. Modaresi, P. Chiquet, S. Stiegeler, C. Verbree, T. Remus, M. Schmitt, C. Kolopp, M. A. Westwood, N. Desjonquères, E. Brabet, S. Hell, K. LePoupon, A. Vermeulen, R. Jaisson, V. Rithié, G. Upert, A. Lederer, P. Zbinden, A. Wach, K. Moehle, K. Zerbe, H. H. Locher, F. Bernardini, G. E. Dale, L. Eberl, B. Wollscheid, S. Hiller, J. A. Robinson, D. Obrecht, Chimeric peptidomimetic antibiotics against Gram-negative bacteria. Nature 576, 452–458 (2019). 31645764 10.1038/s41586-019-1665-6

[7] Y. Imai, K. J. Meyer, A. Iinishi, Q. Favre-Godal, R. Green, S. Manuse, M. Caboni, M. Mori, S. Niles, M. Ghiglieri, C. Honrao, X. Ma, J. J. Guo, A. Makriyannis, L. Linares-Otoya, N. Böhringer, Z. G. Wuisan, H. Kaur, R. Wu, A. Mateus, A. Typas, M. M. Savitski, J. L. Espinoza, A. O’Rourke, K. E. Nelson, S. Hiller, N. Noinaj, T. F. Schäberle, A. D’Onofrio, K. Lewis, A new antibiotic selectively kills Gram-negative pathogens. Nature 576, 459–464 (2019). 31747680 10.1038/s41586-019-1791-1 PMC7188312

[8] E. M. Hart, A. M. Mitchell, A. Konovalova, M. Grabowicz, J. Sheng, X. Han, F. P. Rodriguez-Rivera, A. G. Schwaid, J. C. Malinverni, C. J. Balibar, S. Bodea, Q. Si, H. Wang, M. F. Homsher, R. E. Painter, A. K. Ogawa, H. Sutterlin, T. Roemer, T. A. Black, D. M. Rothman, S. S. Walker, T. J. Silhavy, A small-molecule inhibitor of BamA impervious to efflux and the outer membrane permeability barrier. Proc. Natl. Acad. Sci. U.S.A. 116, 21748–21757 (2019). 31591200 10.1073/pnas.1912345116 PMC6815139

[9] D. J. Sherman, R. Xie, R. J. Taylor, A. H. George, S. Okuda, P. J. Foster, D. J. Needleman, D. Kahne, Lipopolysaccharide is transported to the cell surface by a membrane-to-membrane protein bridge. Science 359, 798–801 (2018). 29449493 10.1126/science.aar1886 PMC5858563

[10] E. Lundstedt, D. Kahne, N. Ruiz, Assembly and maintenance of lipids at the bacterial outer membrane. Chem. Rev. 121, 5098–5123 (2021). 32955879 10.1021/acs.chemrev.0c00587 PMC7981291

[11] P. Sperandeo, A. M. Martorana, A. Polissi, Lipopolysaccharide biogenesis and transport at the outer membrane of Gram-negative bacteria. Biochim. Biophys. Acta Mol. Cell Biol. Lipids 1862, 1451–1460 (2017). 27760389 10.1016/j.bbalip.2016.10.006

[12] S. Mahalakshmi, M. R. Sunayana, L. SaiSree, M. Reddy, yciM is an essential gene required for regulation of lipopolysaccharide synthesis in *Escherichia coli*. Mol. Microbiol. 91, 145–157 (2014). 24266962 10.1111/mmi.12452

[13] R. L. Guest, D. Samé Guerra, M. Wissler, J. Grimm, T. J. Silhavy, YejM modulates activity of the YciM/FtsH protease complex to prevent lethal accumulation of lipopolysaccharide. MBio 11, e00598-20 (2020). 32291302 10.1128/mBio.00598-20 PMC7157816

[14] T. Clairfeuille, K. R. Buchholz, Q. Li, E. Verschueren, P. Liu, D. Sangaraju, S. Park, C. L. Noland, K. M. Storek, N. N. Nickerson, L. Martin, T. dela Vega, A. Miu, J. Reeder, M. Ruiz-Gonzalez, D. Swem, G. Han, D. P. DePonte, M. S. Hunter, C. Gati, S. Shahidi-Latham, M. Xu, N. Skelton, B. D. Sellers, E. Skippington, W. Sandoval, E. J. Hanan, J. Payandeh, S. T. Rutherford, Structure of the essential inner membrane lipopolysaccharide–PbgA complex. Nature 584, 479–483 (2020). 32788728 10.1038/s41586-020-2597-x

[15] P. Sperandeo, F. K. Lau, A. Carpentieri, C. de Castro, A. Molinaro, G. Dehò, T. J. Silhavy, A. Polissi, Functional analysis of the protein machinery required for transport of lipopolysaccharide to the outer membrane of *Escherichia coli*. J. Bacteriol. 190, 4460–4469 (2008). 18424520 10.1128/JB.00270-08 PMC2446812

[16] S. Vetterli, K. Zerbe, M. Müller, M. Urfer, M. Mondal, S.-Y. Wang, K. Moehle, O. Zerbe, A. Vitale, G. Pessi, L. Eberl, B. Wollscheid, J. A. Robinson, Thanatin targets the intermembrane protein complex required for lipopolysaccharide transport in *Escherichia coli*. Sci. Adv. 4, eaau2634 (2018). 30443594 10.1126/sciadv.aau2634 PMC6235536

[17] E. C. C. M. Moura, T. Baeta, A. Romanelli, C. Laguri, A. M. Martorana, E. Erba, J. P. Simorre, P. Sperandeo, A. Polissi, Thanatin impairs lipopolysaccharide transport complex assembly by targeting LptC–LptA interaction and decreasing LptA stability. Front. Microbiol. 11, 909 (2020). 32477309 10.3389/fmicb.2020.00909 PMC7237710

[18] P. Fehlbaum, P. Bulet, S. Chernysh, J. P. Briand, J. P. Roussel, L. Letellier, C. Hetru, J. A. Hoffmann, Structure-activity analysis of thanatin, a 21-residue inducible insect defense peptide with sequence homology to frog skin antimicrobial peptides. Proc. Natl. Acad. Sci. U.S.A. 93, 1221–1225 (1996). 8577744 10.1073/pnas.93.3.1221 PMC40060

[19] F. Fiorentino, J. B. Sauer, X. Qiu, R. A. Corey, C. K. Cassidy, B. Mynors-Wallis, S. Mehmood, J. R. Bolla, P. J. Stansfeld, C. V. Robinson, Dynamics of an LPS translocon induced by substrate and an antimicrobial peptide. Nat. Chem. Biol. 17, 187–195 (2021). 33199913 10.1038/s41589-020-00694-2 PMC7116625

[20] C. Laguri, P. Sperandeo, K. Pounot, I. Ayala, A. Silipo, C. M. Bougault, A. Molinaro, A. Polissi, J. P. Simorre, Interaction of lipopolysaccharides at intermolecular sites of the periplasmic Lpt transport assembly. Sci. Rep. 7, 9715 (2017). 28852068 10.1038/s41598-017-10136-0 PMC5575297

[21] N. Mandard, P. Sodano, H. Labbe, J. M. Bonmatin, P. Bulet, C. Hetru, M. Ptak, F. Vovelle, Solution structure of thanatin, a potent bactericidal and fungicidal insect peptide, determined from proton two-dimensional nuclear magnetic resonance data. Eur. J. Biochem. 256, 404–410 (1998). 9760181 10.1046/j.1432-1327.1998.2560404.x

[22] T. Imamura, N. Yamamoto, A. Tamura, S. Murabayashi, S. Hashimoto, H. Shimada, S. Taguchi, NMR based structure-activity relationship analysis of an antimicrobial peptide, thanatin, engineered by site-specific chemical modification: Activity improvement and spectrum alteration. Biochem. Biophys. Res. Commun. 369, 609–615 (2008). 18294955 10.1016/j.bbrc.2008.02.057

[23] S.Sinha, V. B. Dhanabal, P.Sperandeo, A. Polissi, S. Bhattacharjya, Linking dual mode of action of host defense antimicrobial peptide thanatin: Structures, lipopolysaccharide and LptAm binding of designed analogs. Biochim. Biophys. Act. (BBA) - Biomembranes 1864, 183839 (2022). 10.1016/j.bbamem.2021.183839 34915021

[24] K. M. Schultz, C. S. Klug, Characterization of and lipopolysaccharide binding to the E. coli LptC protein dimer. Protein Sci. 27, 381–389 (2018). 29024084 10.1002/pro.3322 PMC5775163

[25] K. M. Schultz, M. A. Fischer, E. L. Noey, C. S. Klug, Disruption of the E. coli LptC dimerization interface and characterization of lipopolysaccharide and LptA binding to monomeric LptC. Protein Sci. 27, 1407–1417 (2018). 29672978 10.1002/pro.3429 PMC6153404

[26] C. Hobson, A. N. Chan, G. D. Wright, The antibiotic resistome: A guide for the discovery of natural products as antimicrobial agents. Chem. Rev. 121, 3464–3494 (2021). 33606500 10.1021/acs.chemrev.0c01214

[27] P. Zhou, A. A. Lugovskoy, G. Wagner, A solubility-enhancement tag (SET) for NMR studies of poorly behaving proteins. J. Biomol. NMR 20, 11–14 (2001). 11430750 10.1023/a:1011258906244

[28] M. Schuster, M. Deluigi, M. Pantić, S. Vacca, C. Baumann, D. J. Scott, A. Plückthun, O. Zerbe, Optimizing the α_1B_-adrenergic receptor for solution NMR studies. Biochim. Biophys. Acta Biomembr. 1862, 183354 (2020). 32413443 10.1016/j.bbamem.2020.183354

[29] M. Sattler, J. Schleucher, C. Griesinger, Heteronuclear multidimensional NMR experiments for the structure determination of proteins in solution employing pulsed field gradients. Prog. Nucl. Magn. Reson. Spectrosc. 34, 93–158 (1999).

[30] G. Otting, K. Wüthrich, Heteronuclear filters in two-dimensional (^1^H,^1^H) NMR spectroscopy: Combined use with isotope labelling for studies of macromolecular conformation and intermolecular interactions. Q. Rev. Biophys. 23, 39–96 (1990). 2160666 10.1017/s0033583500005412

[31] P. Güntert, Automated NMR structure calculation with CYANA. Methods Mol. Biol. 278, 353–378 (2004). 15318003 10.1385/1-59259-809-9:353

[32] Y. Shen, A. Bax, Protein backbone and sidechain torsion angles predicted from NMR chemical shifts using artificial neural networks. J. Biomol. NMR 56, 227–241 (2013). 23728592 10.1007/s10858-013-9741-y PMC3701756

[33] H. Bekker, H. J. C. Berendsen, E. J. Dijkstra, S. Achterop, R. Vondrumen, D. Vanderspoel, A. Sijbers, H. Keegstra, M. K. R. Renardus, Gromacs–A parallel computer for molecular-dynamics simulations. Phys. Comput. Secur. '92, 252–256 (1993).

[34] R. B. Best, X. Zhu, J. Shim, P. E. M. Lopes, J. Mittal, M. Feig, A. D. MacKerell Jr., Optimization of the additive CHARMM all-atom protein force field targeting improved sampling of the backbone ϕ, ψ and side-chain χ1and χ2 dihedral angles. J. Chem. Theor. Comput. 8, 3257–3273 (2012). 10.1021/ct300400x PMC3549273 23341755

