# Supplementary material for: Peptidomimetic antibiotics disrupt the lipopolysaccharide transport bridge of drug-resistant Enterobacteriaceae
Source: Sci Adv. 2023 May 24;9(21):eadg3683. doi: 10.1126/sciadv.adg3683 (PMC10208570; doi:10.1126/sciadv.adg3683)
Supplement: Supplementary file 1 — Figs. S1 to S27 Tables S1 to S13 [file sciadv.adg3683_sm.pdf]

Science Advances
AAAS

Supplementary Materials for

Peptidomimetic antibiotics disrupt the lipopolysaccharide transport bridge of drug-resistant Enterobacteriaceae

Matthias Schuster *et al.*

Corresponding author: Daniel Obrecht, daniel.obrecht@spexisbio.com;
Mohammed Benghezal, mbenghezal@gmail.com; Oliver Zerbe, oliver.zerbe@chem.uzh.ch

Sci. Adv. **9**, eadg3683 (2023)
DOI: 10.1126/sciadv.adg3683

This PDF file includes:

Figs. S1 to S27
Tables S1 to S13

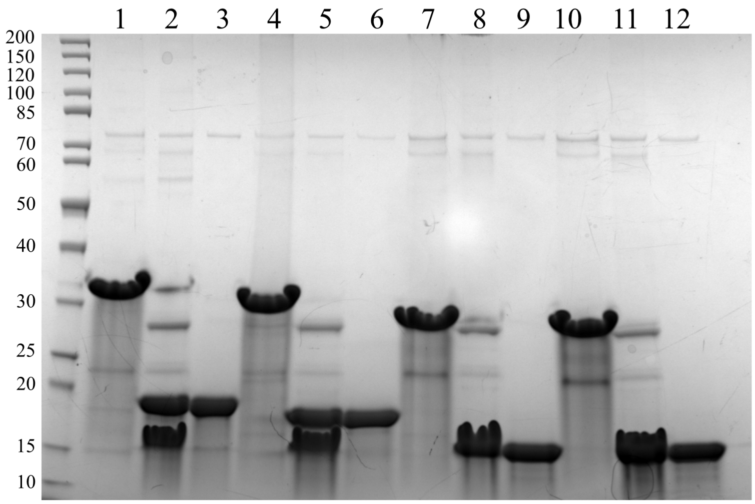

1 GB1-LptC^{AA}
2 GB1-LptC^{AA} +TEV
3 LptC^{AA}
4 GB1-mLptA
5 GB1-mLptA +TEV
6 mLptA
7 GB1-LptAm
8 GB1-LptAm +TEV
9 LptAm
10 GB1-LptAm^{Q62L}
11 GB1-LptAm^{Q62L} +TEV
12 LptAm^{Q62L}

Fig. S1. SDS-PAGE of *E. coli* Lpt proteins at various stages of purification.

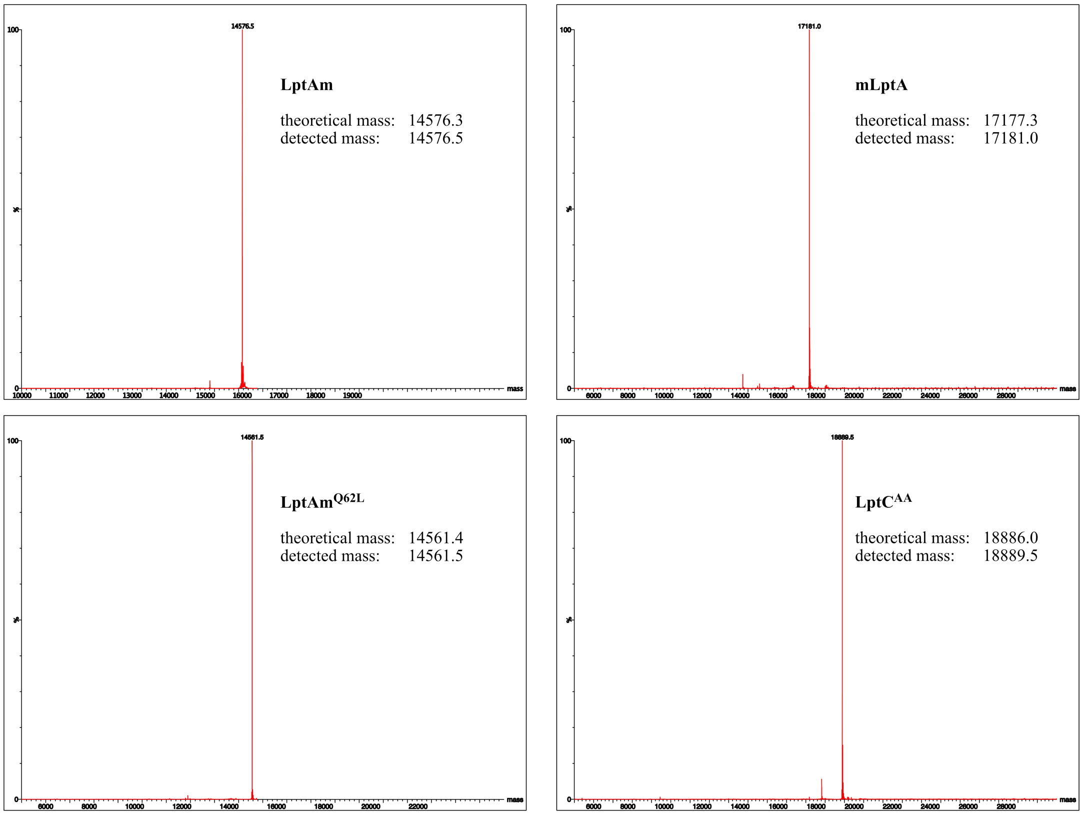

Fig. S2. ESI-MS of the *E. coli* Lpt proteins.

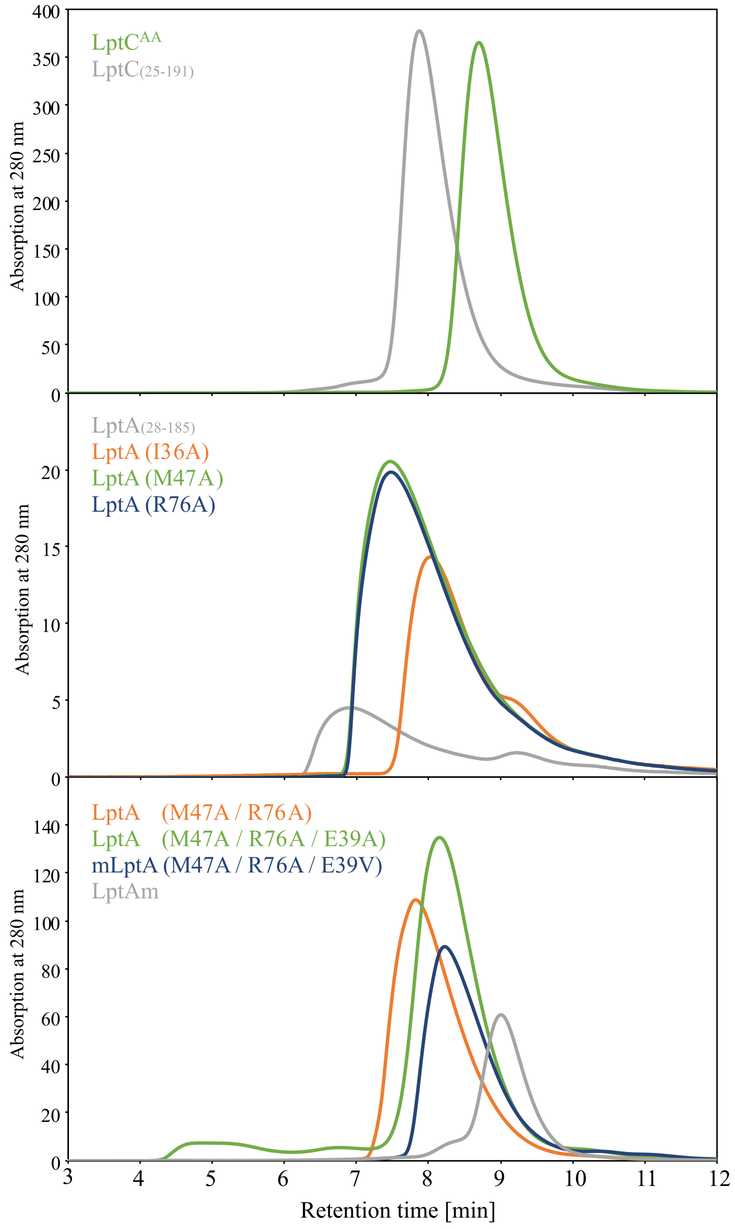

Fig. S3. SEC chromatograms of *E. coli* LptA and LptC and their mutants to reduce oligomerization. Size exclusion chromatography (SEC) was performed on a Superdex Increase 5/150 or 10/300 column (Cytiva) in SEC Buffer (20 mM Na-Phosphate pH 7, 150 mM NaCl) on an Agilent 1260 Infinity System with a flow rate of 0.25 mL/min or 0.5 mL/min respectively. 10 or 80 µL of the protein sample was loaded with the Agilent Infinity Autosampler. The multi-angle light-scattering (MALS) was measured with a Wyatt miniDAWN TEREOS and a Shodex RI-101 detector. Curves were analysed with the ASTRA 6 software and masses were calculated. For the proteins, a standard dn/dc value of 0.185 was used. $LptC^{AA}$ corresponds to a monomeric version of LptC, $LptC^{Y60A, R61A}$.

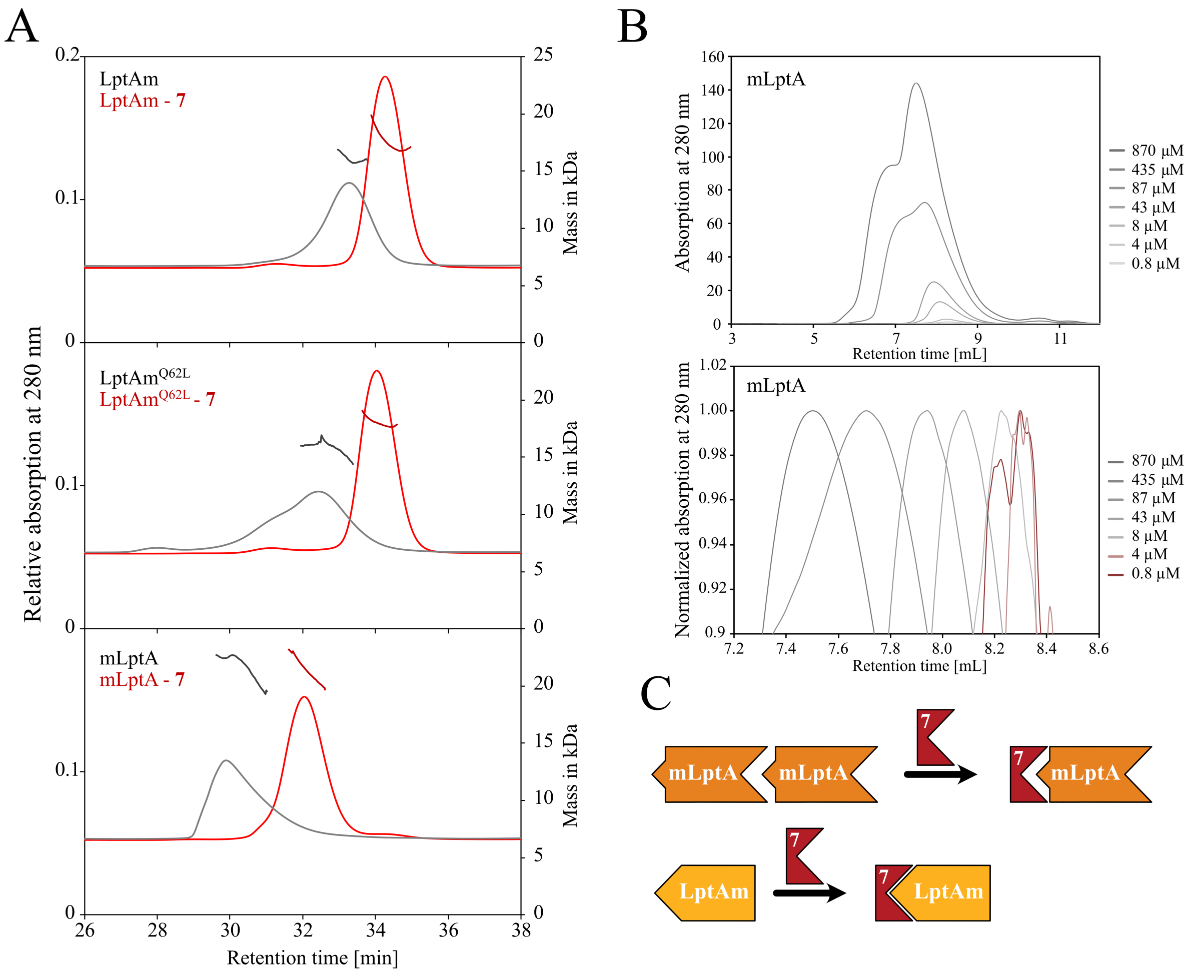

Fig. S4. SEC-MALS analysis of *E. coli* Lpt monomers (A) SEC-MALS analysis of LptA with and without **7**. LptAm and LptAm^{Q62L} show an increased mass of about 2 kDa after binding of the peptide. In contrast, the retention time increases after binding to the peptide due to a more compact fold and a smaller hydrodynamic radius. The measured mass of mLptA is slightly larger in the unbound state compared to its monomeric mass. (B) mLptA is still weakly interacting with itself to form larger homo-oligomers. At concentrations lower than 4 µM the peak maximum is not shifting anymore, suggesting a Kd in this concentration range. (C) Schematic representation of the binding.

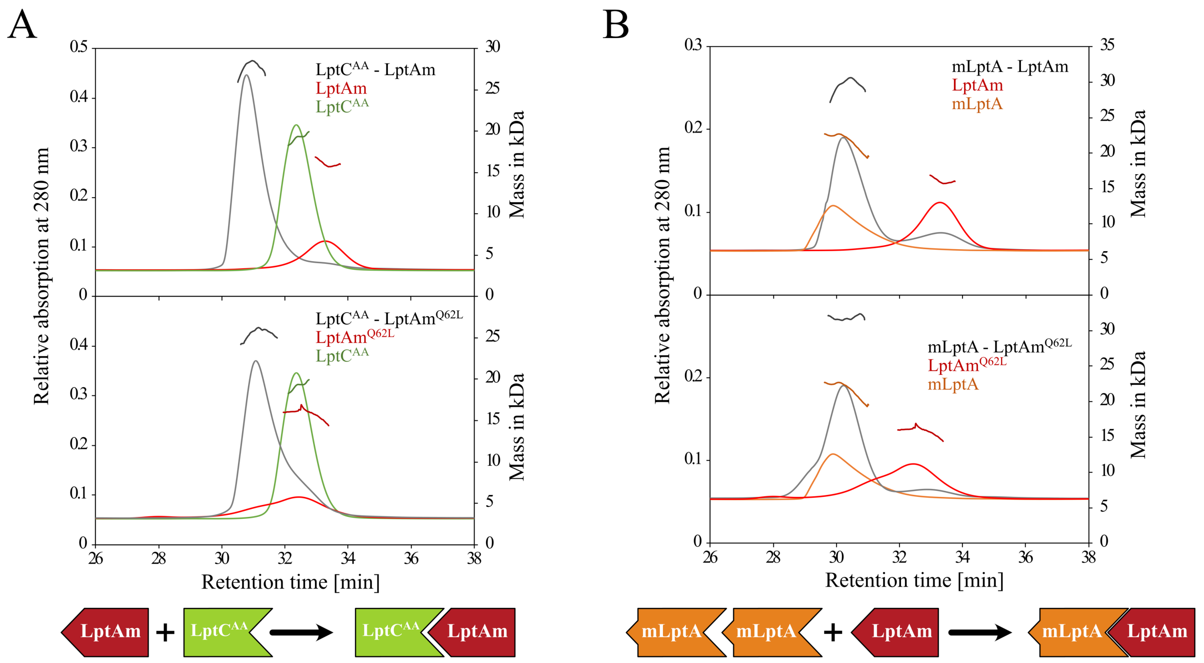

Fig. S5. SEC-MALS analysis of *E. coli* Lpt dimers (A) SEC-MALS analysis of the LptAm-LptC^{AA} and (B) the LptAm-mLptA interactions. The mass of the heterodimer is increasing while the retention time is decreasing in comparison to the monomers.

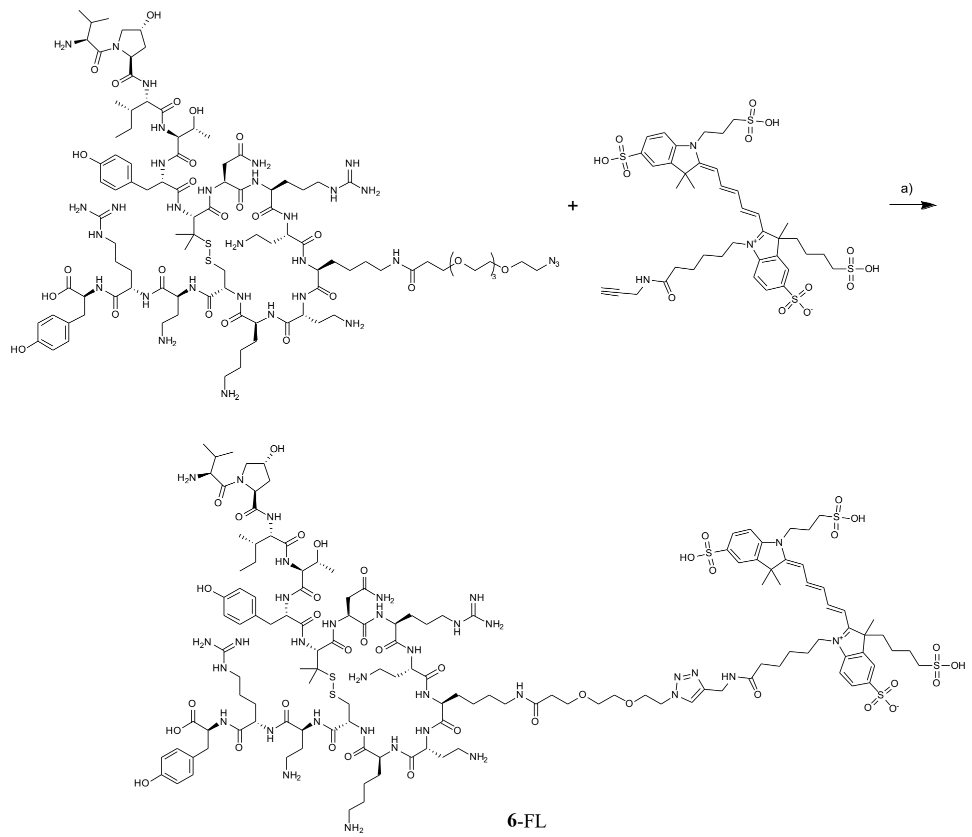

6-FL

Fig. S6. Synthesis of 6-FL. The peptide precursor was assembled by solid-phase synthesis on 2-chlorotrityl resin using Fmoc chemistry. a) $CuSO_4 * 5H_2O$, ascorbic acid, MeOH/water.

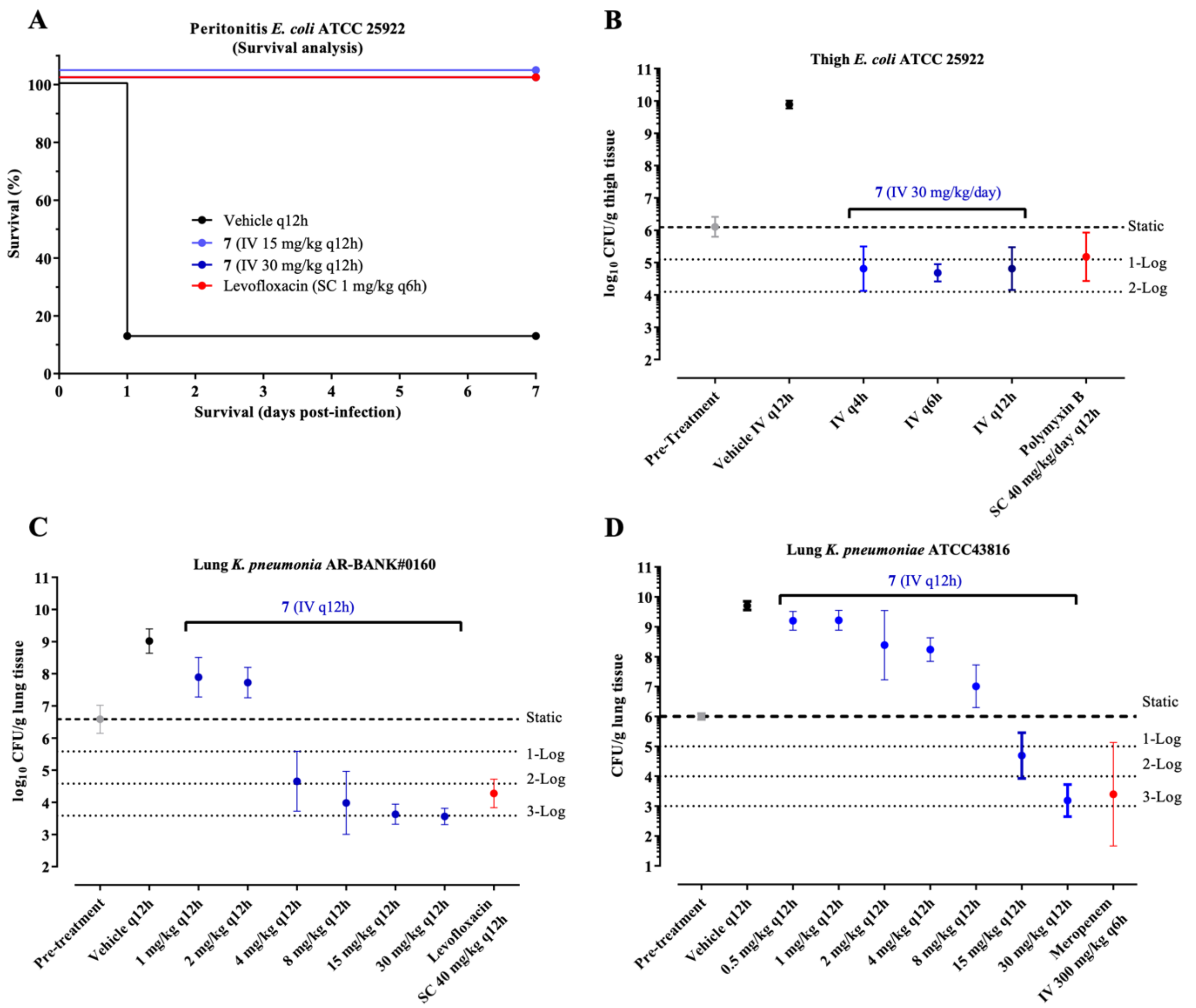

Fig. S7. ***In vivo*** **efficacy of peptide 7 in different infection models in neutropenic mice. (A)** Kaplan-Meier plot survival rate in *E. coli* ATCC 25922 peritonitis model (Fisher's exact test, $p < 0.05$). **(B)** Efficacy in *E. coli* ATCC 25922 thigh model (non-parametric Kruskal-Wallis test, $p < 0.0001$), **(C)** Efficacy in *K. pneumoniae* AR-BANK#0160 lung model (one-way ANOVA followed by Dunnett's test, $p < 0.05$). **(D)** Efficacy in *K. pneumoniae* ATCC 43816 lung model (non-parametric Kruskal-Wallis test, $p < 0.0001$). Compound **7** efficacy (blue) is compared to the administration of vehicle only (black) and standard of care (red). The geometric mean value and standard deviation of each group is depicted.

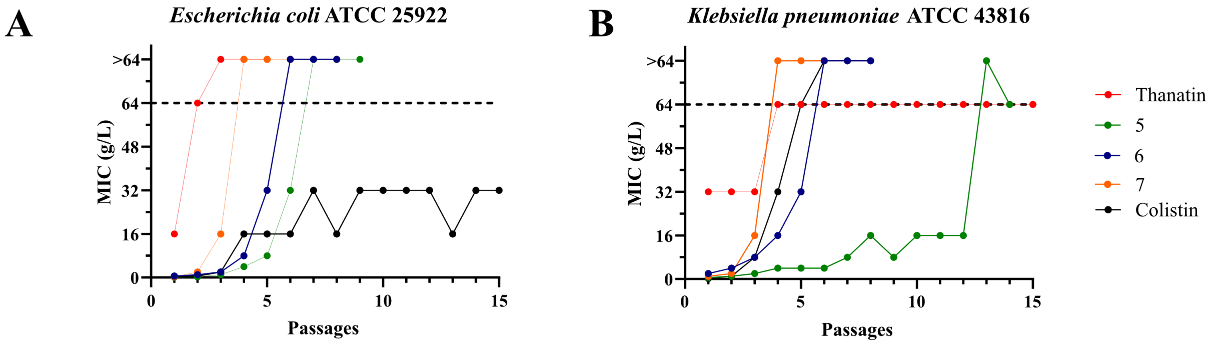

Fig. S8. Resistance development studies by serial passage. Broth containing a single antibiotic at sub-MIC and multiples of the MIC were inoculated with bacteria. Drug concentrations were increased during passaging in case bacteria were growing at a higher drug concentration. *Both E. coli* (A) and *K. pneumoniae* (B) species developed resistance against thanatin (red) rapidly after day 1 and 2 of passaging. The thanatin derivatives **6** (blue), **7** (orange), and **5** (green) developed resistance more slowly between days 3 and 8 days for *E. coli* and 6 to >15 for *K. pneumoniae*. As a benchmark, *E. coli* developed resistance against colistin (black) from day 5 to 7 whereas *K. pneumoniae* developed resistance against colistin very rapidly after day 1 to 3.

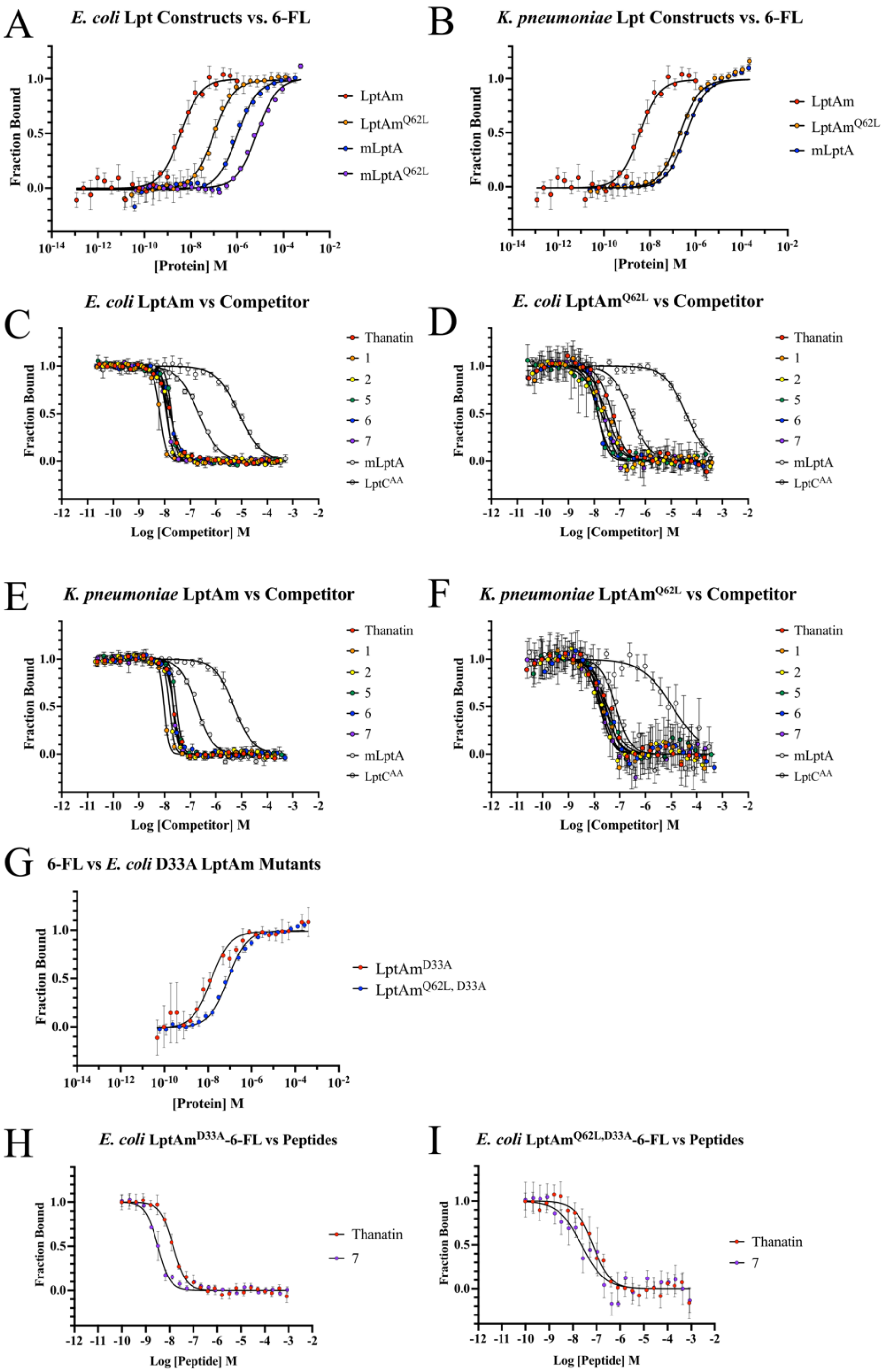

Fig. S9. Representative curves from FP data. Data are shown for *E. coli* (A,C,E) and *K. pneumoniae* (B,D,F) Lpt proteins. (A,B) Direct FP binding curves for **6**-FL interactions. (C,D) Indirect assays for LptAm-**6**-FL. (E,F) Indirect assays for LptAm^{Q62L}-**6**-FL. (G) Direct binding of **6**-FL to EC-LptAm^{D33A}. (H) Indirect assay for **6**-FL against EC-LptAm^{D33A} or EC-LptAm^{Q62L,D33A} (I). All FP curves were measured in triplicates and error bars represent the standard deviation.

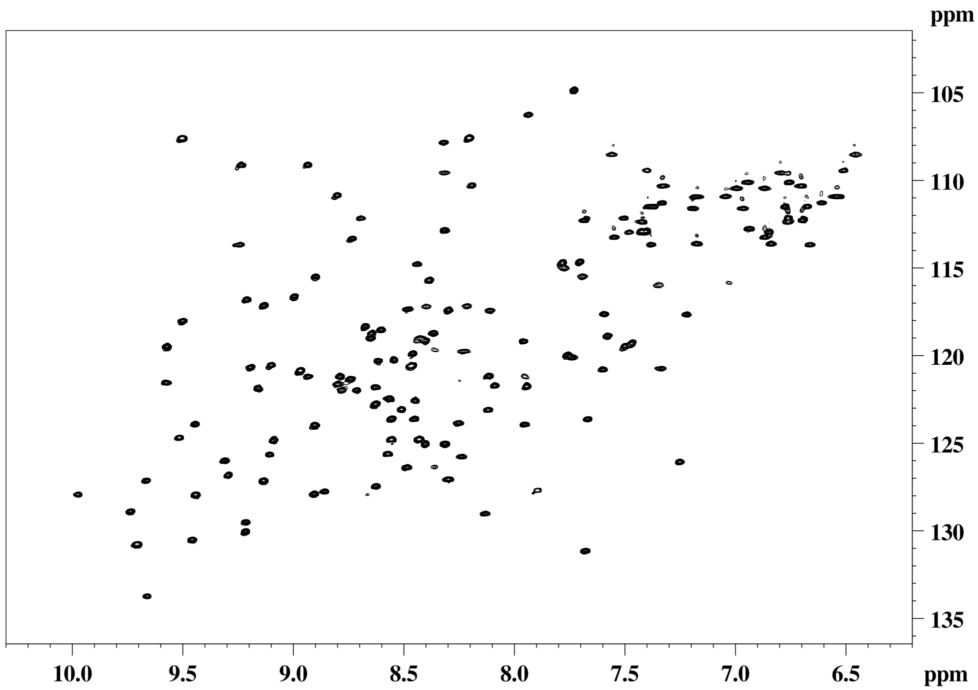

Fig. S10. [^{15}N,^{1}H]-HSQC of 200 μM *E. coli* LptAm complexed to **7**, measured at 600 MHz, 298 K.

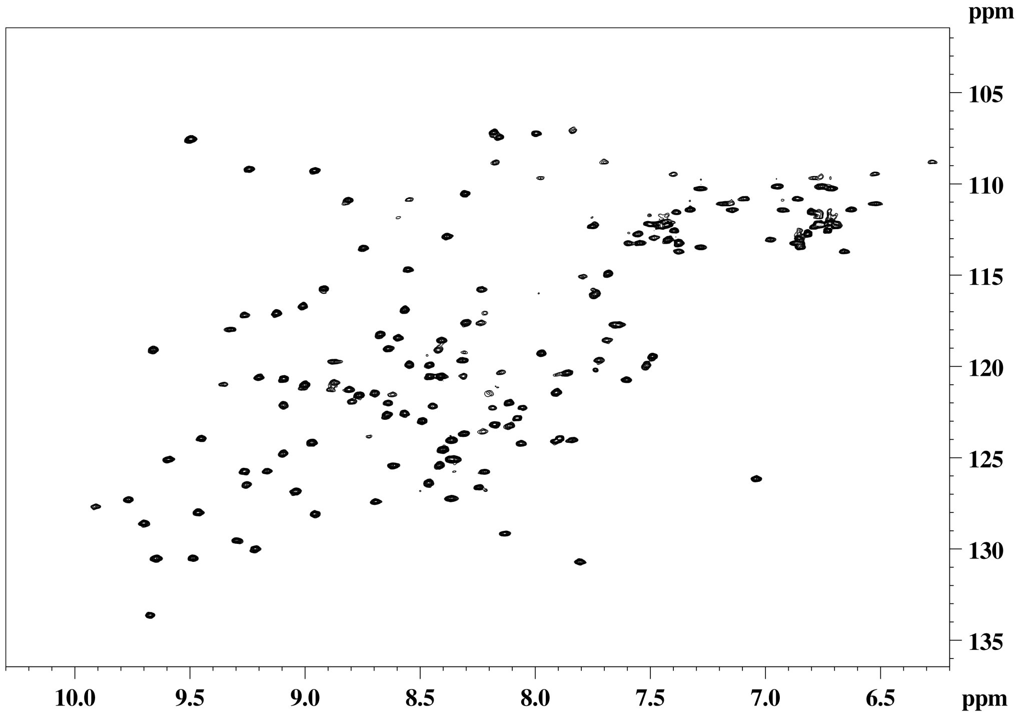

Fig. S11. [^{15}N,^{1}H]-HSQC of 200 µM *E. coli* mLptA complexed to **7**, measured at 600 MHz, 310 K.

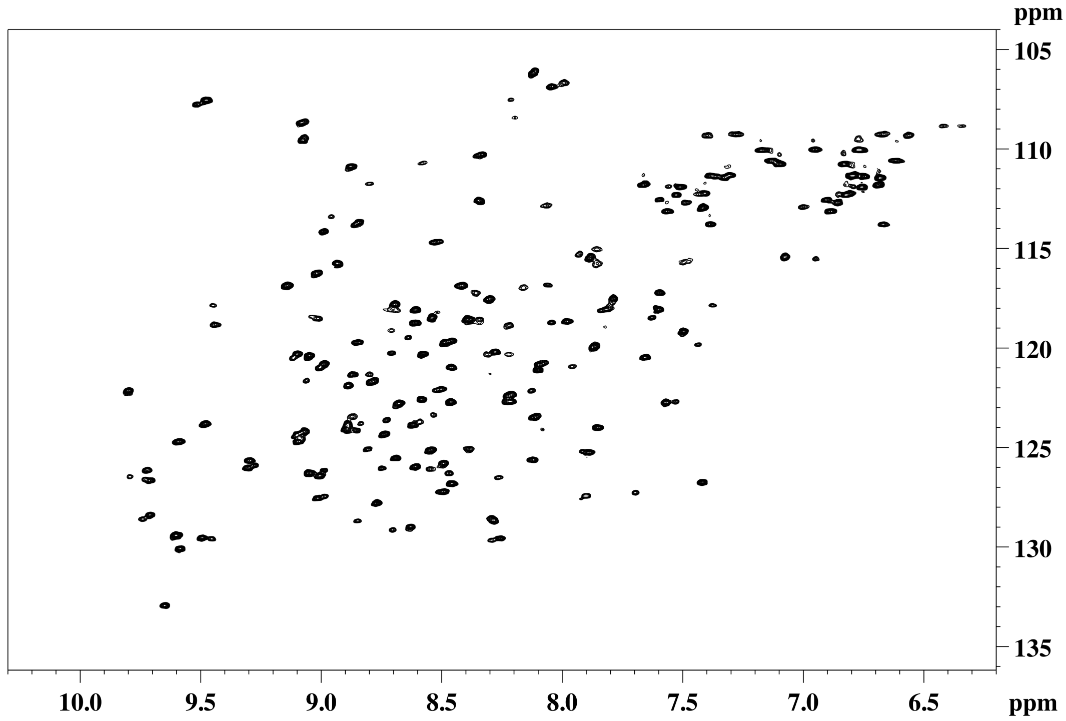

Fig. S12. [^{15}N,^{1}H]-HSQC of 200 μM *E. coli* LptAm^{Q62L} complexed to **7**, measured at 600 MHz, 298 K.

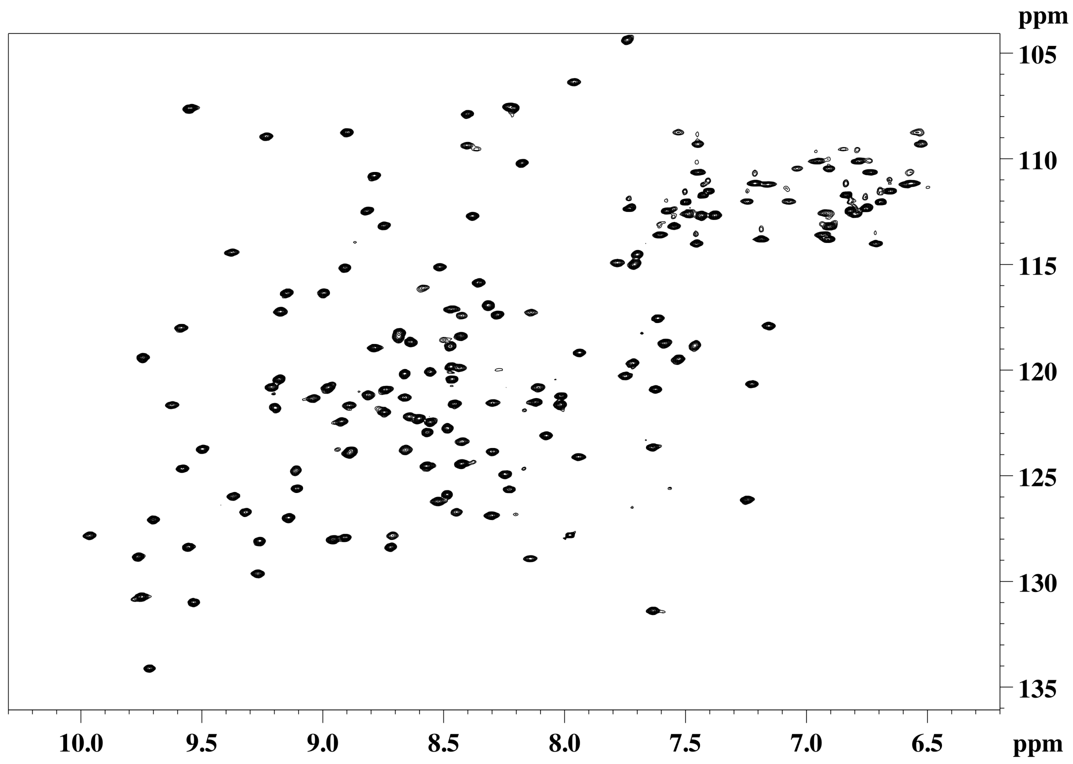

Fig. S13. [^{15}N,^{1}H]-HSQC of 200 μM *E. coli* LptAm complexed to **5**, measured at 700 MHz, 298 K.

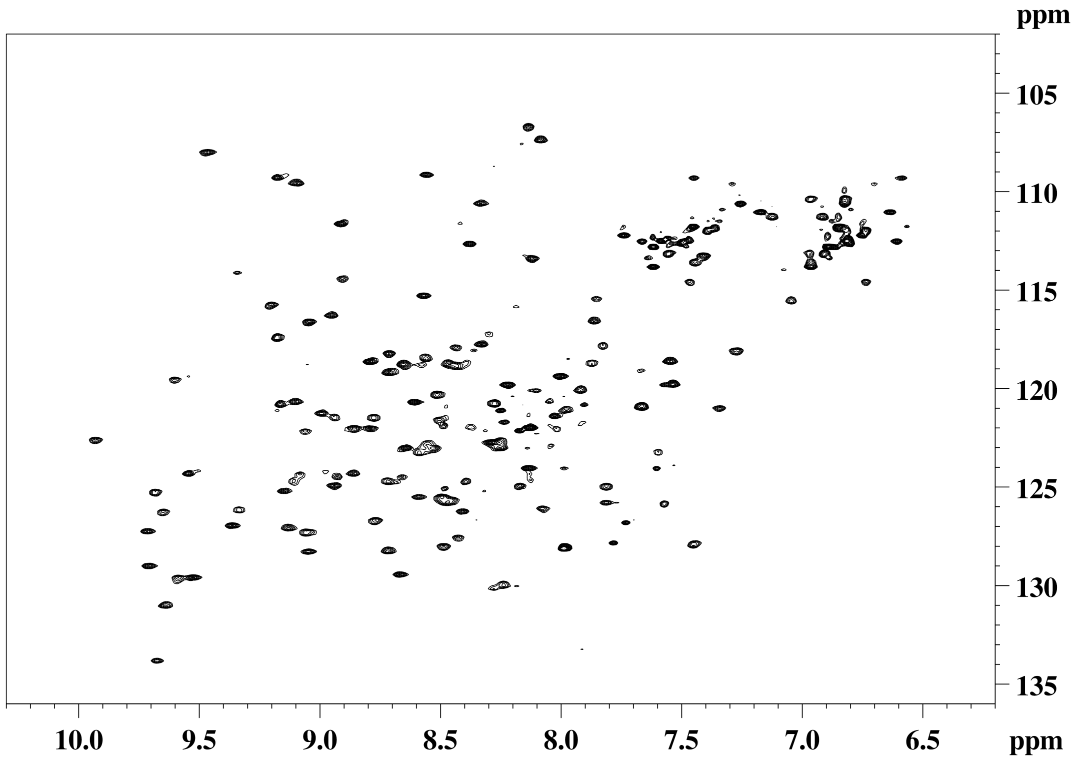

Fig. S14. [^{15}N,^{1}H]-HSQC of 200 μM *E. coli* LptAm^{Q62L} complexed to **5**, measured at 600 MHz, 298 K.

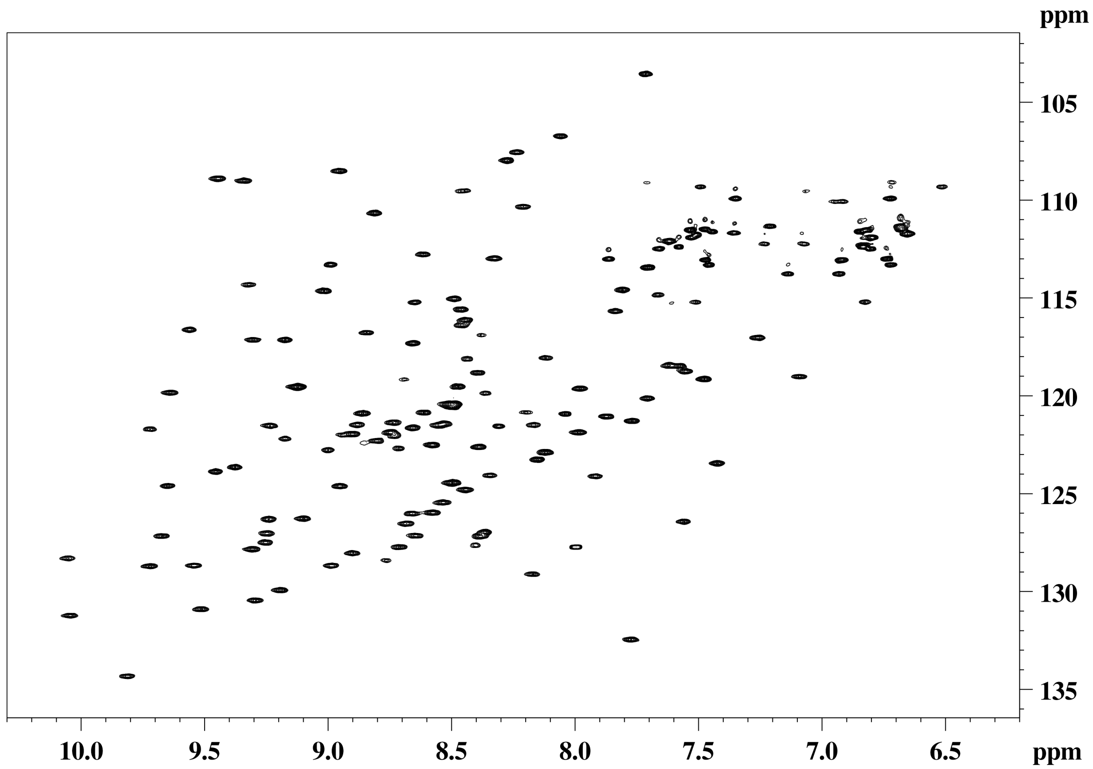

Fig. S15. [^{15}N,^{1}H]-HSQC of 200 μM *K. pneumoniae* LptAm complexed to **7**, measured at 600 MHz, 298 K.

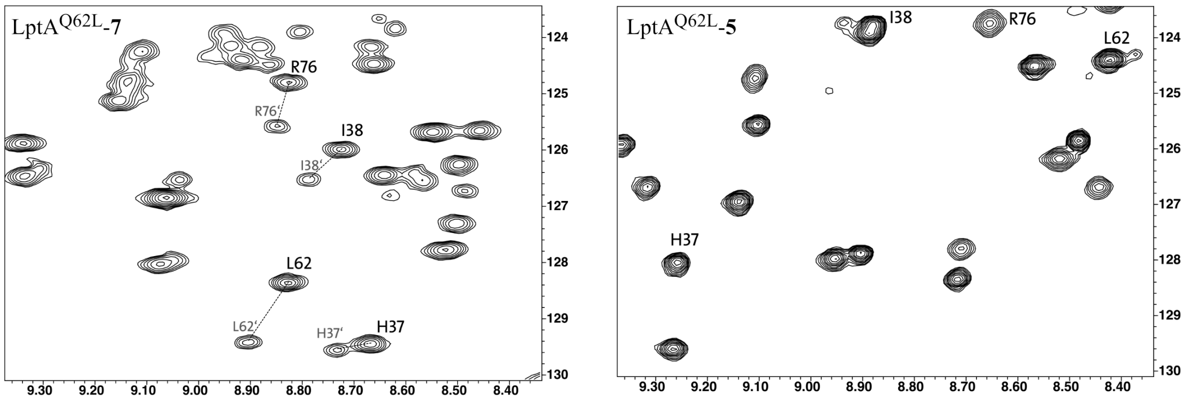

Fig. S16. Comparison of a selected region of the [^{15}N,^{1}H]-HSQC of 200 μM *E. coli* LptA^{Q62L} complexed to **7** (left) and **5** (right), measured at 600 MHz, 298 K. Peak-doubling in the LptA^{Q62L}-**7** complex is indicated by dotted lines. A second set of peaks is visible in presence of **7** due to cis/trans isomerism at the V6'-Hyp7' bond not visible in presence of the tighter binder **5**.

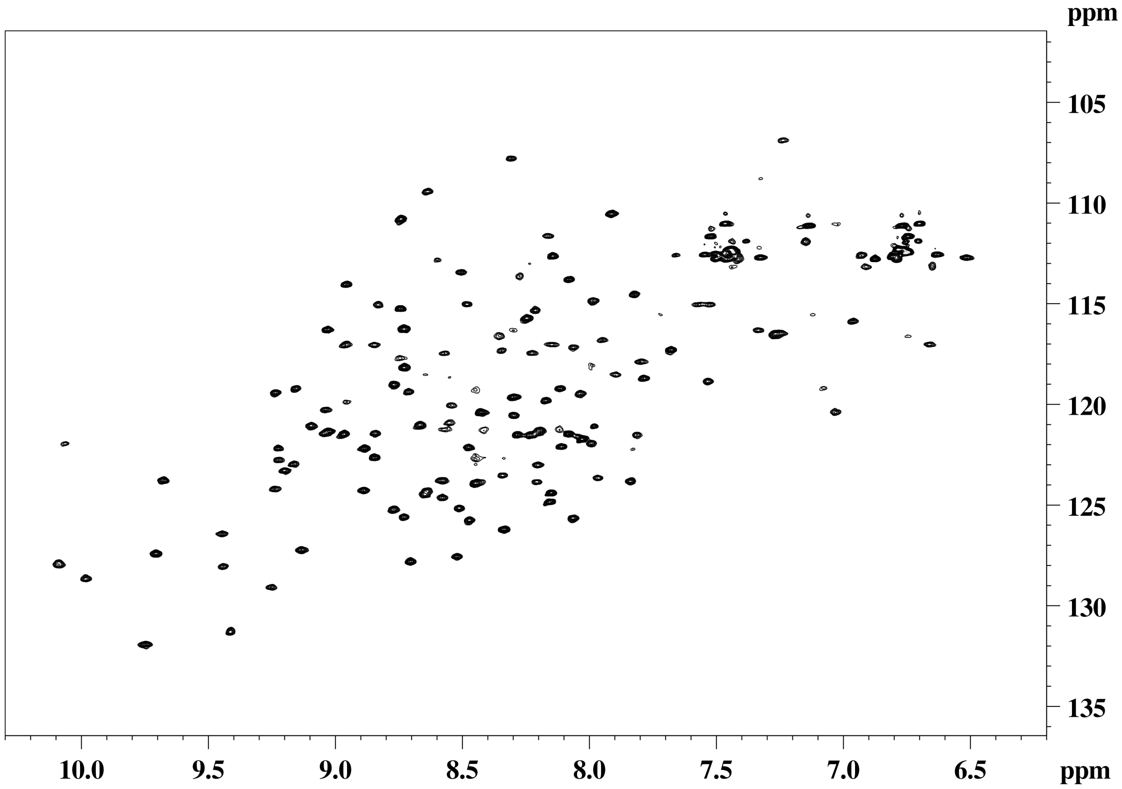

Fig. S17. [^{15}N,^{1}H]-HSQC of 200 μM *E. coli* LptC^{AA}, measured at 600 MHz, 310 K.

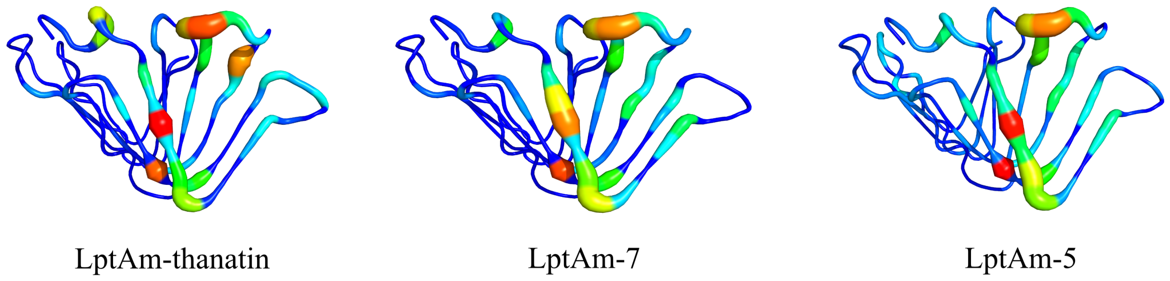

Fig. S18. Chemical shift perturbations (CSPs) of *E. coli* LptAm upon addition of thantin (left), **7** (center) or **5** (right) mapped on the LptAm structure. The radius of the spline function is proportional to the CSPs. In addition, the magnitude of CSPs is color-coded (green-to-red in the range 0.05-1 ppm)

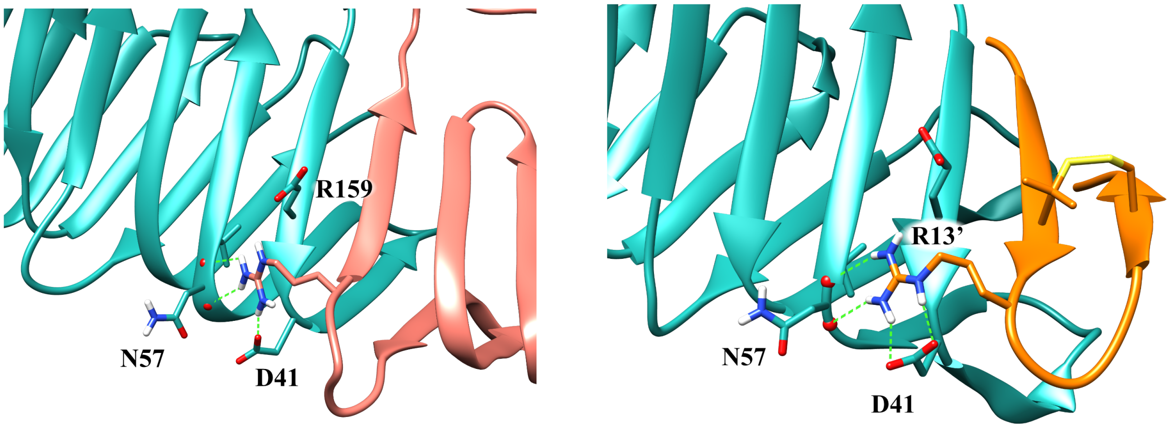

Fig. S19. Comparison of the binding interface in the *E. coli* LptA-LptA dimer (left) and in the *E. coli* LptAm-**7** complex (right).

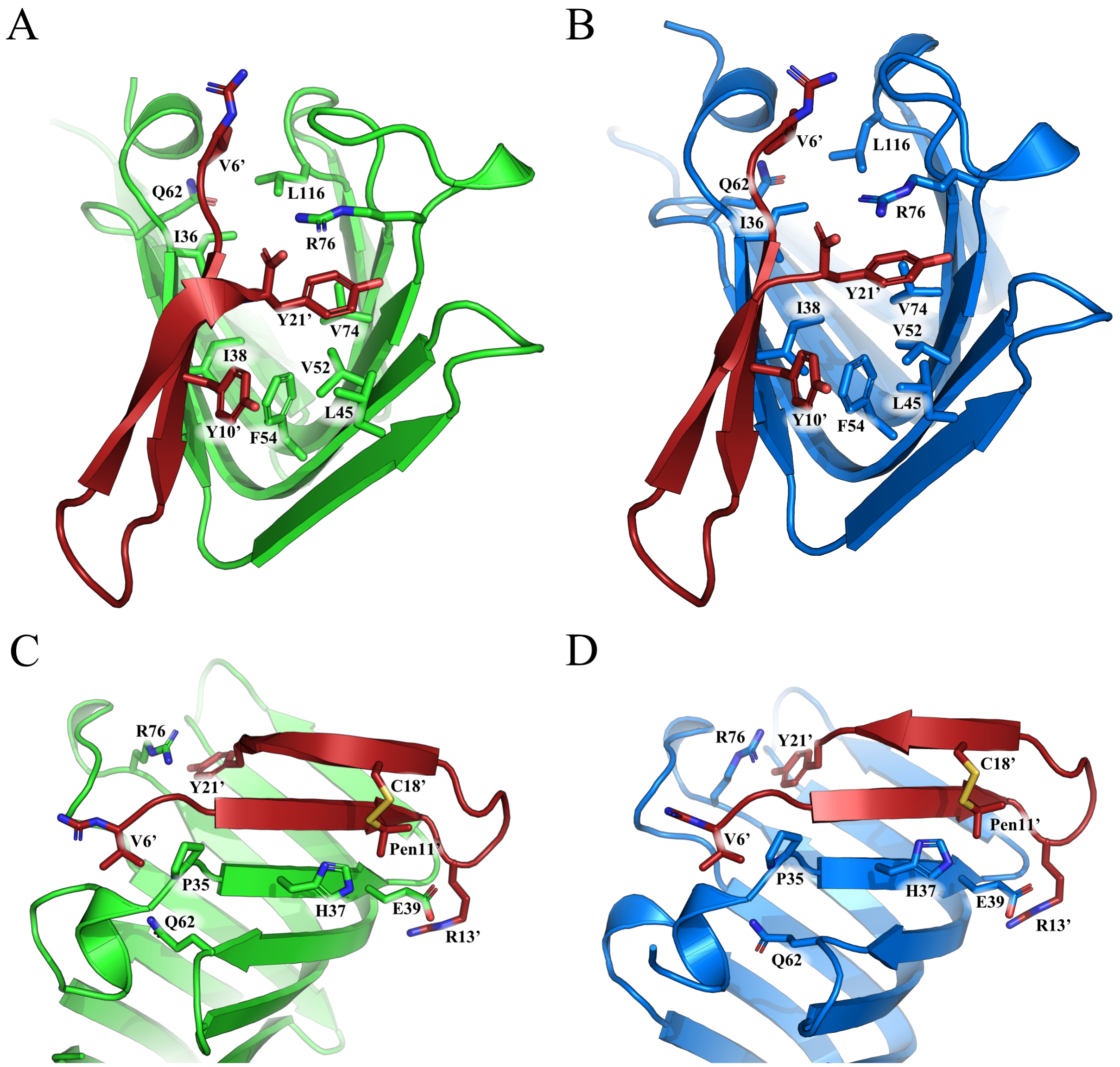

Fig. S20. Comparison of compound **7** (red) binding to the N-terminus of LptAm in *E. coli* (in green, **A**, **C**) and *K. pneumoniae* (in blue, **B**, **D**). Sidechains of residues forming important interactions are labeled. **A** and **B** observe the binding interface from the top, highlighting important side chain residues—forming mainly van der Waals contacts—inward facing with the peptide. **C** and **D** observe the binding interface from the side, highlighting important side chain residues outward facing that interact with the peptide.

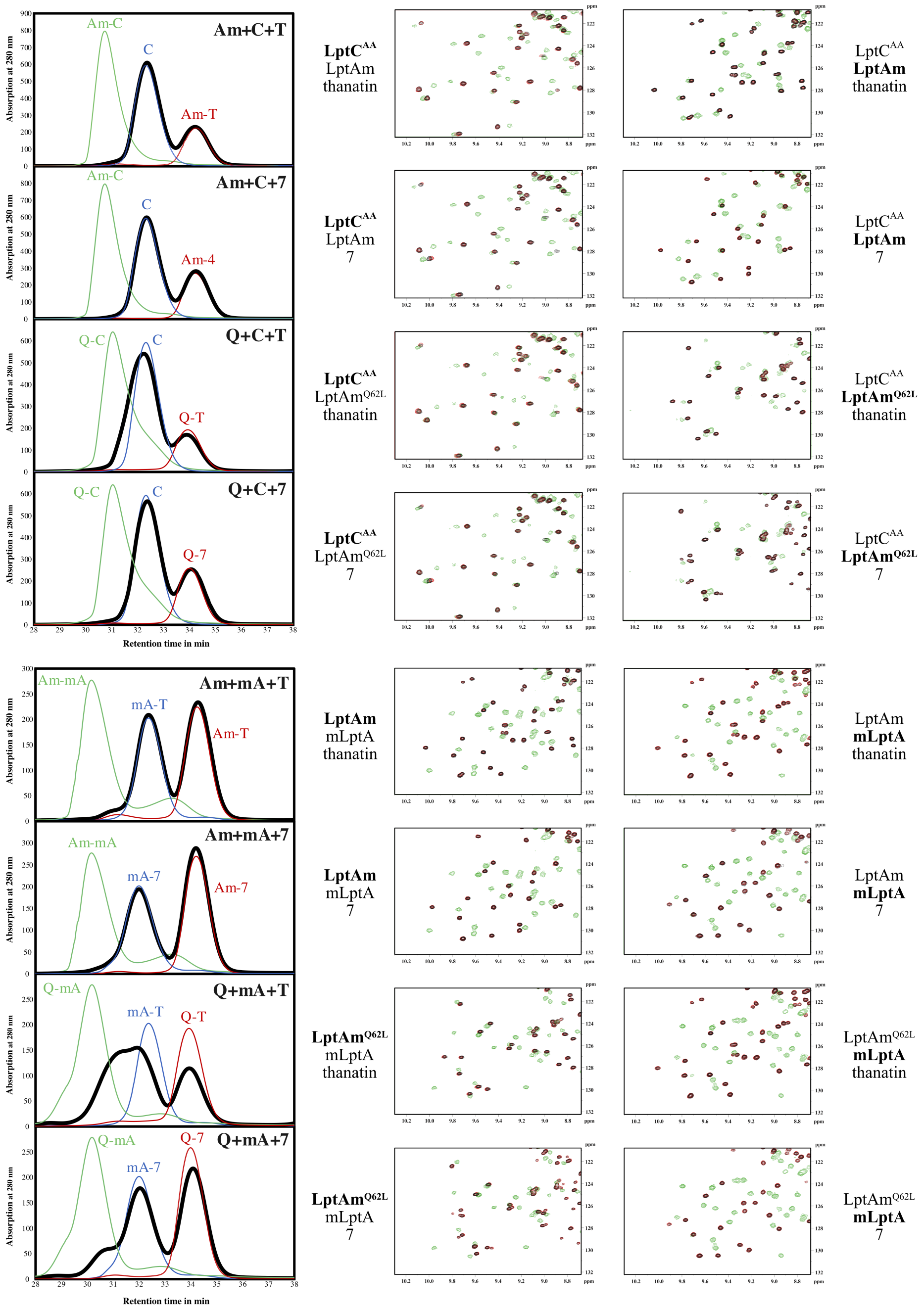

Fig. S21. Disassembly probed by SEC and NMR. SEC traces of the mixture of two *E. coli* proteins (mLptA-LptAm and LptAm-LptC^{AA} and the corresponding complexes with LptAm^{Q62L}) and either thanatin or **7**. A short notation for Lpt proteins is used for clarity (A=LptA, C=LptC^{AA}, Q=LptAm^{Q62L} and T=Thanatin). Next to it are shown the corresponding $[^{15}N,^{1}H]$-HSQC spectra overlays with the ^{15}N labeled protein depicted with bold font. The green peaks are from the protein-protein dimers. The red peaks are from the protein-peptide complex alone. The black peaks are from the protein-protein-peptide mixture. For both NMR and SEC experiments, the protein concentration was always 200 μM while the peptide added was 1.5 equivalents.

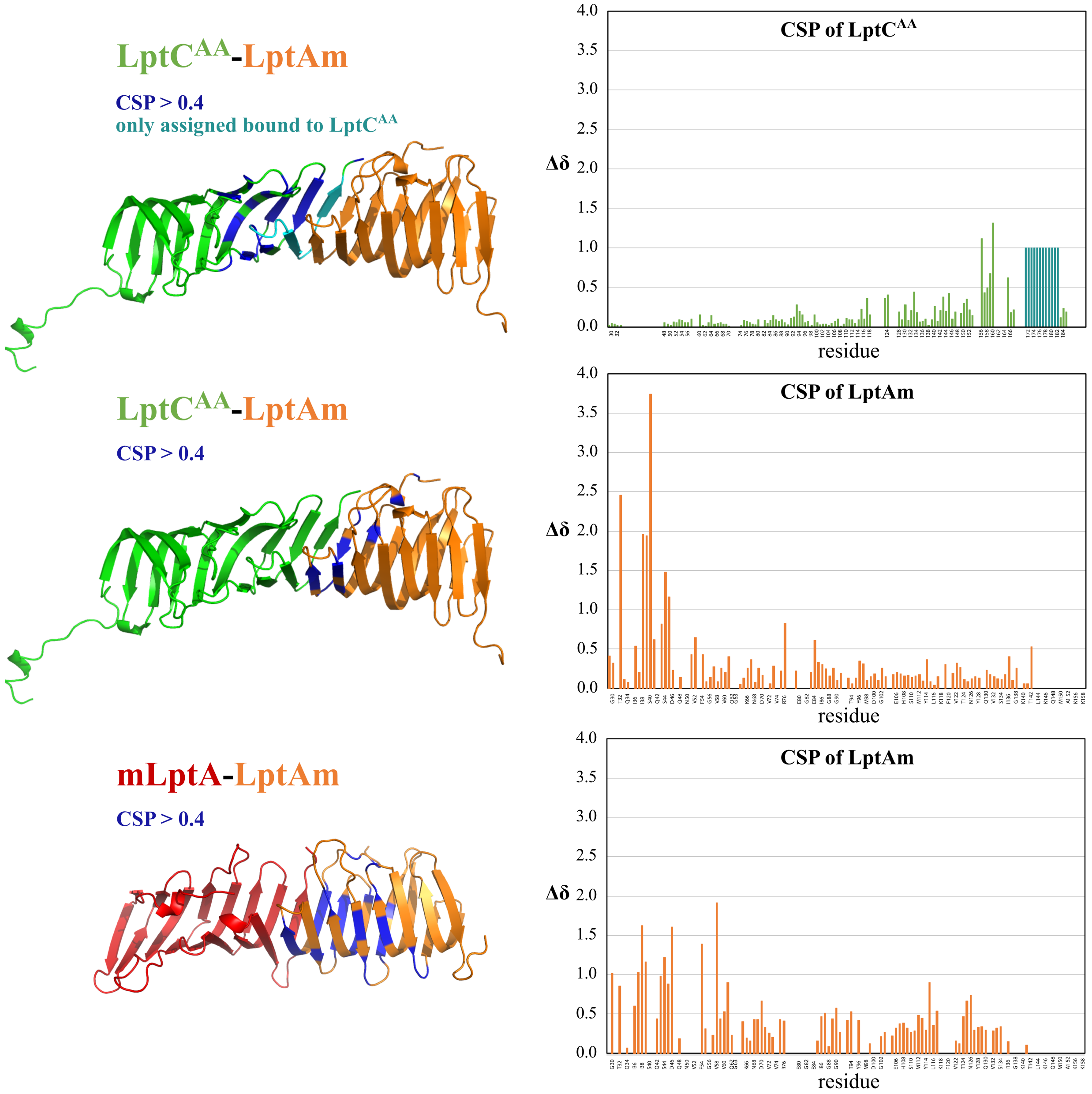

Fig. S22. Chemical shift perturbations (CSP) of *E. coli* LptC^{AA} when complexed to LptAm (top), *E. coli* LptAm bound to LptC^{AA} (center), and *E. coli* LptAm bound to mLptA (bottom). Shifts bigger than 0.4 are color-coded in blue on a structure model (left) or presented as values for every residue (right).

The largest CSPs were observed in the C-terminal part of LptC$^{AA.}$ The last two beta strands could only be assigned in complex with LptAm, presumably due to signal broadening from accelerated amide exchange. We therefore concluded that the LptAm binding site involves these two C-terminal beta strands of LptC. Additionally, LptAm displays the strongest CSPs in the first 2 N-terminal beta strands confirming the suggested binding mode. In complex with mLptA the LptAm shifts are more evenly distributed over the entire protein.

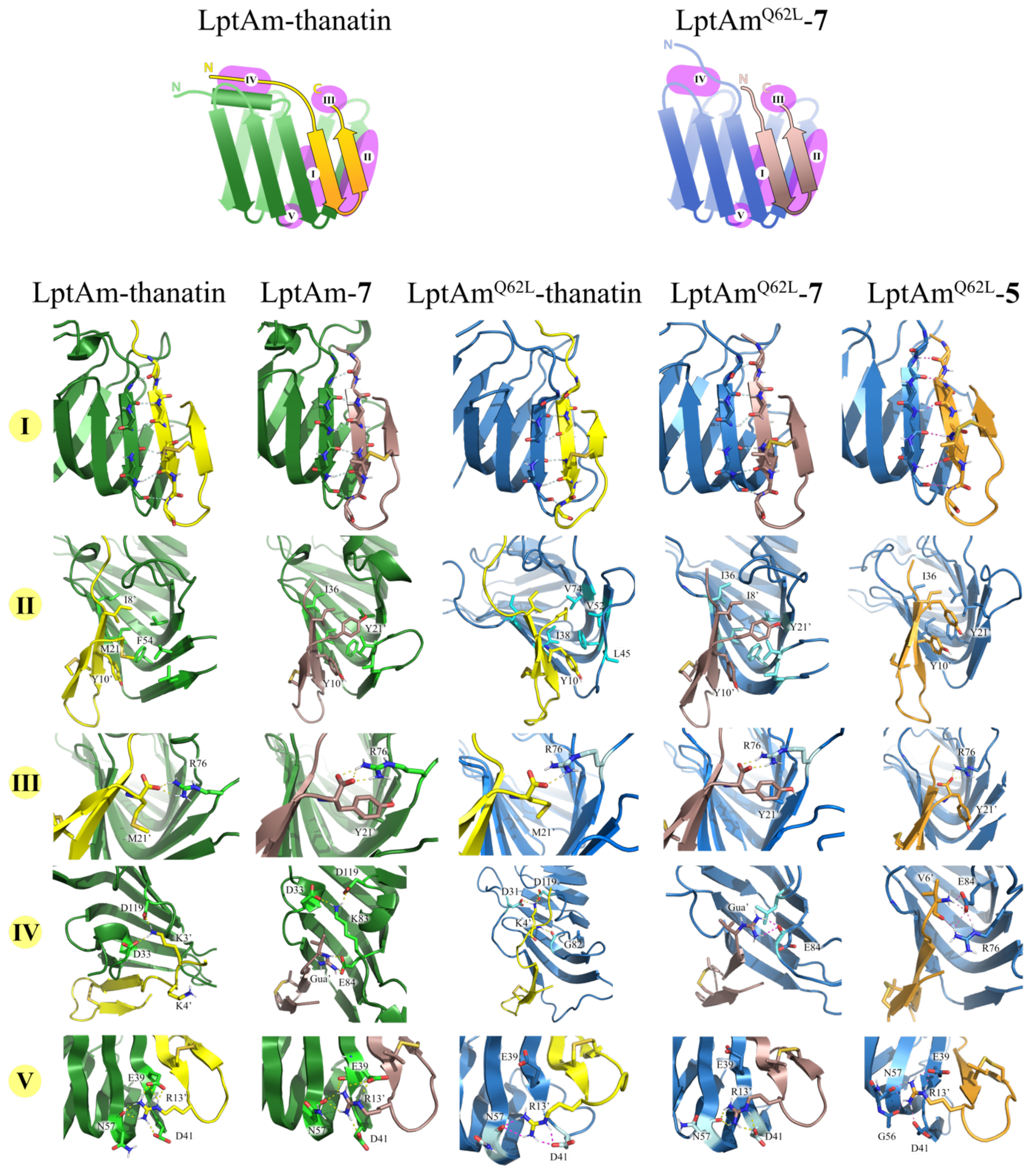

Fig. S23. Structural details of the *E. coli* protein complexes with peptides obtained as averages during MD-simulation starting from the lowest-energy NMR structures. Depicted are details of thanatin, **5** or **7** in their complexes with LptAm and LptAm^{Q62L} in sites I-V. The structure of the LptAm^{Q62L}-thanatin complex is derived from an MD simulation based on a model derived from the LptAm-thanatin complex.

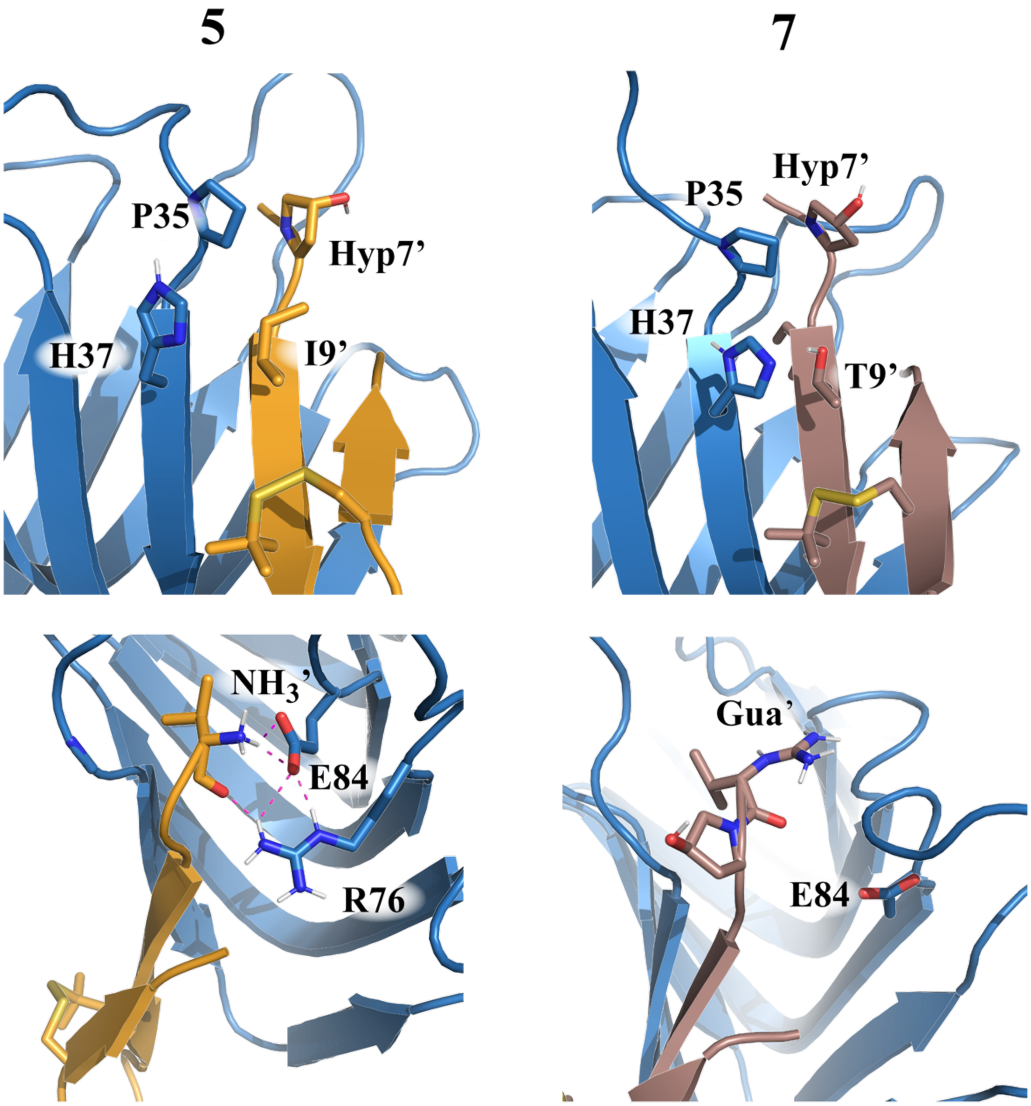

Peptide Residue										
Peptide	6-VAL	7-HYP	8-ILE	9-ILE/THR	10-TYR	11-PEN	12-ASN	13-ARG	21-TYR	Total
**5**	6	2	11	10	8	6	1	1	7	52
**7**	2	0	0	0	10	7	1	2	8	30

Fig. S24. Comparison of binding modes of 5 and 7 in complex with *E. coli* LptAm^{Q62L}. Depicted are sites I (top) and IV (bottom) for **5** (left) and **7** (right). The number of interfacial NOEs are shown at the bottom.

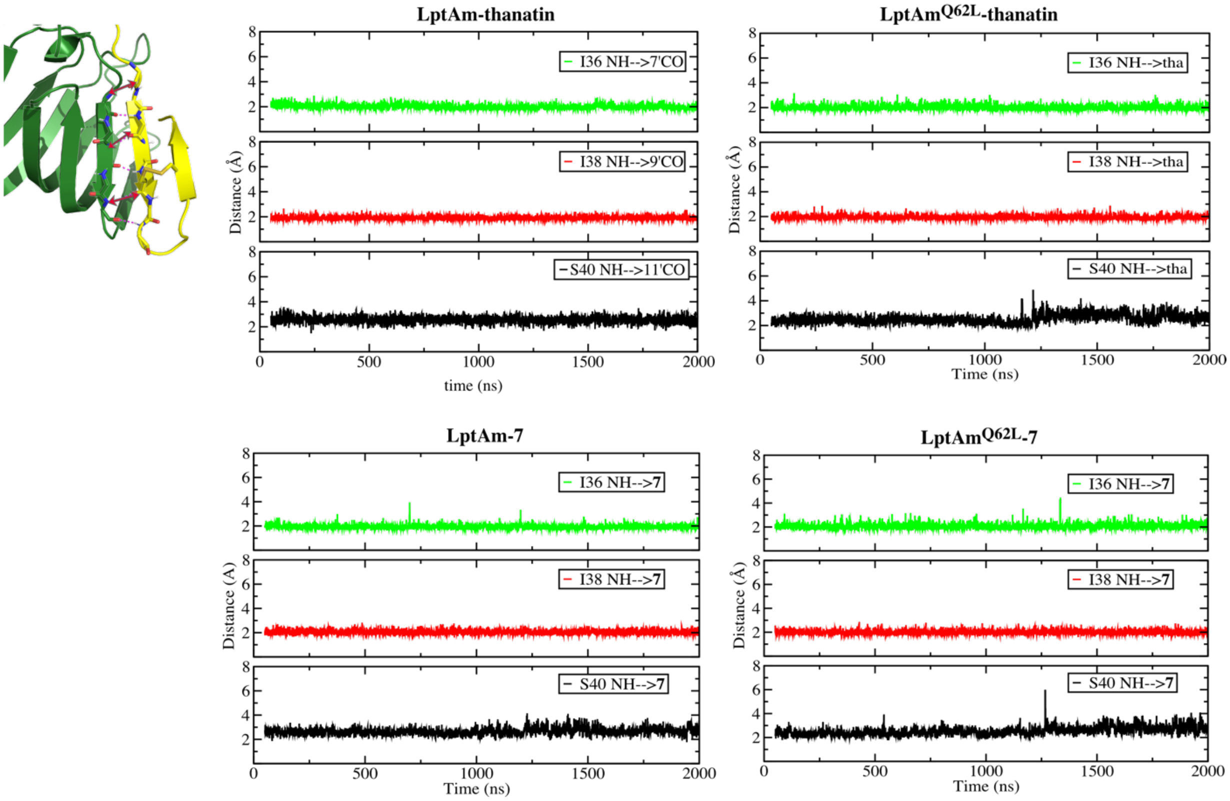

Fig. S25. Minimum distances as a function of time during the MD simulations of complexes characteristic of backbone hydrogen bonds between the peptides and the first beta strand of the β-jellyroll (Site 1). Data are depicted for LptAm (left) and LptAm^{Q62L} (right) for thanatin (top) and **7** (bottom).

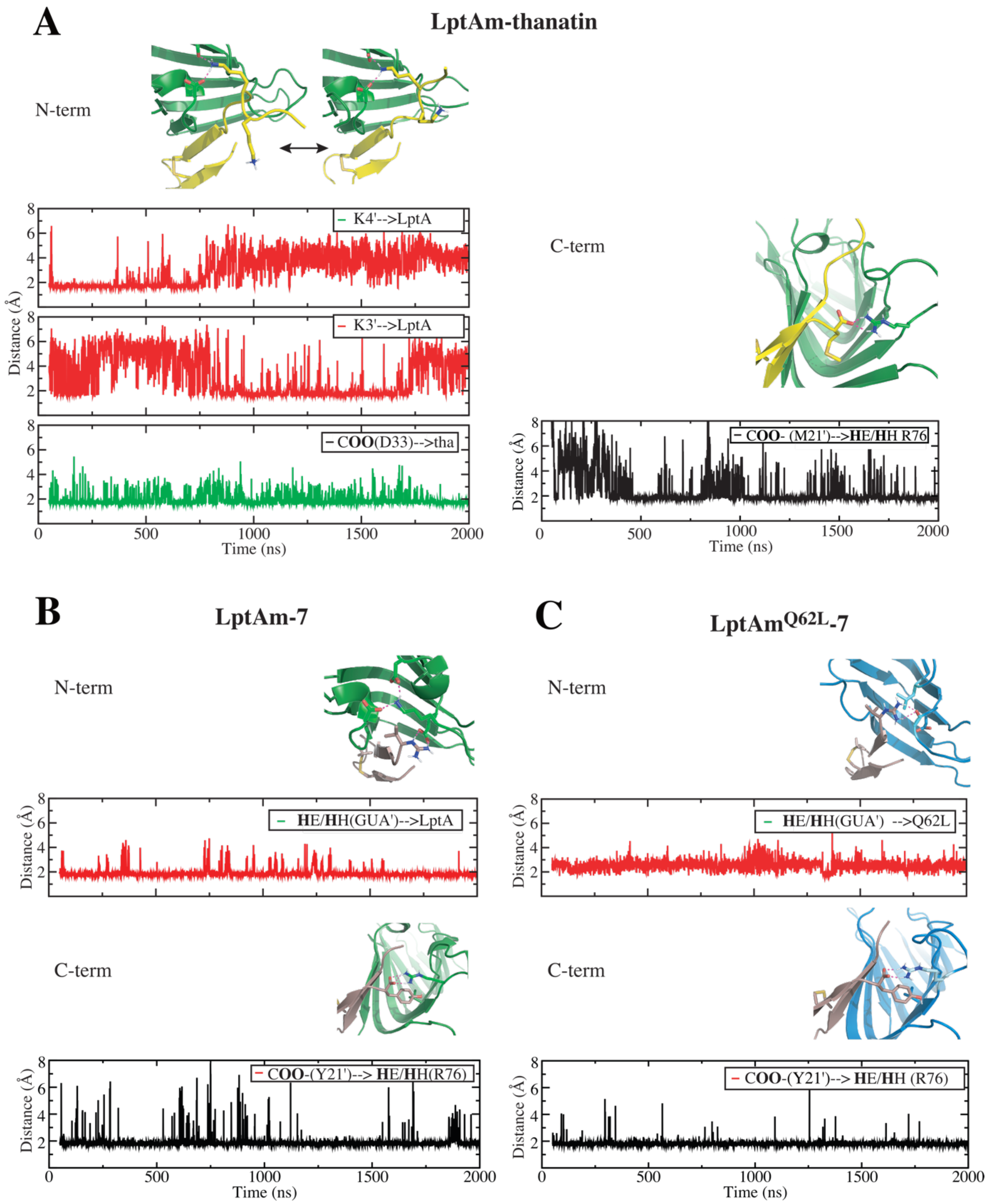

Fig. S26. Minimum distances as a function of time during the MD simulations (Sites 2 or 3). Top left: Distances corresponding to contacts of the N-terminal part of thanatin with LptAm. Top right: Distances of C terminus of thanatin to LptAm. Bottom left: Distances of N or C terminus of **7** with LptAm. Bottom right: Distances of N or C terminus of **7** with LptAm^{Q62L}. Distances are measured between specific atoms (in bold face), when specified, or to closest atom from that residue (or protein).

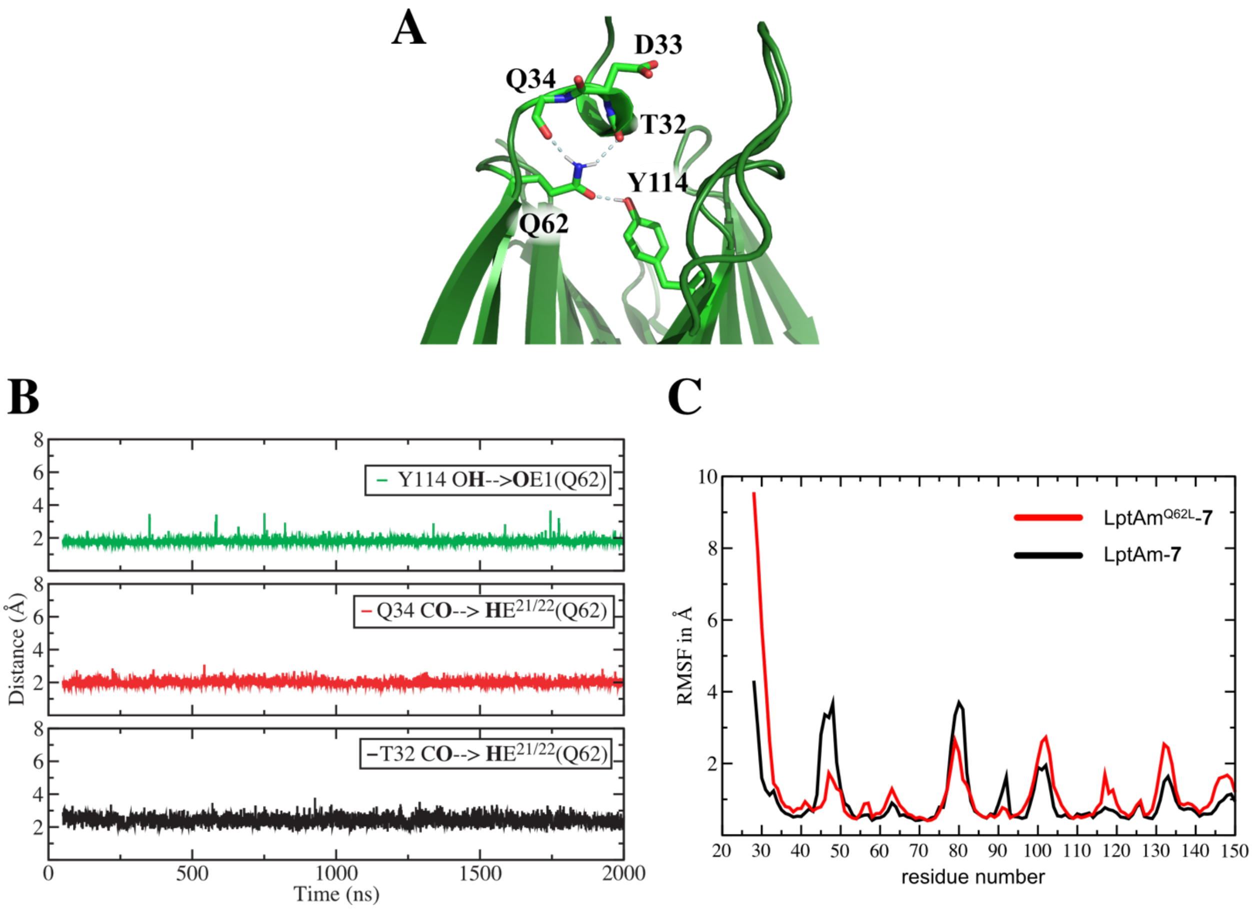

Fig. S27. Selected distances from MD simulations involving Q62. (A) Important intramolecular contacts involving Q62. **(B)** Distances in *E. coli* LptAm during a 2 μs MD trajectory. **(C)** Root-mean square fluctuations (RMSF) per residues during the 2 μs MD trajectory for *E. coli* LptAm (black) and LptAm^{Q62L} (red) in complex with **7**.

LptAm V28 SapI fwd	TTTGCTCTTCGCAGGGAGTAACCGGAGATACTGATCAG
LptAm R159 BamHI rev	TTTGGATCCTTATTAGCGCTTGCCTTTGTCGC
LptA N185 BamHI rev	TTTGGATCCTTATTAATTACCCTTCTTCTGTGCCGGGG
LptC A25 SapI fwd	TTTGCTCTTCGCAGGGAGCCGAAAAAGACGATACCG
LptC P191 BamHI rev	GCAGCCGGATCCTTATTAAGGCTGAGTTTGTTTG
LptC YRAA fwd	CAGAAGGGGCACTAAGCGCTGCATTGATTGCTCAACAC GTTG
LptC YRAA rev	CGTGTTGAGCAATCAATGCAGCGCTTAGTGCCCCTTCTG GG
LptA R76A fwd	AAAGTGGTCGTTACCGCTCCGGGCGGCGAACAAGG
LptA R76A rev	TGTTCGCCGCCCGGAGCGGTAACGACCACTTTGTC
LptA M47A fwd	CAGCAATCTCTTGATGCGCAAGGCAACGTGGTTACC
LptA M47A rev	AACCACGTTGCCTTGCGCATCAAGAGATTGCTGGTC
LptA V28 E39V SapI fwd	TTTGCTCTTCGCAGGGAGTAACCGGAGATACTGATCAGC CGATCCACATTGTATCGGACC
LptA V28 D33A SapI fwd	TTTGCTCTTCGCAGGGAGTAACCGGAGATACTGCTCAGC CGATCC
Kp LptA K28 SapI fwd	TTTGCTCTTCGCAGGGAAAGACGGGCGATACCGATC
Kp LptAm R159 BamHI rev	TTTGGATCCTTATTAGCGTCGACCTTTGTCGC
Kp LptA Q62L fwd	CGTGGTCGTGACCCTGGGGACGATCAAGATTAAC
Kp LptA Q62L rev	CTTGATCGTCCCCAGGGTCACGACCACGTTACCG
Kp mLptA gBlock	TTTGCTCTTCGCAGGGAAAGACGGGCGATACCGATCAG CCGATCCATATCGTGTCCGATCAGCAGTCGCTGGACGCG CAGGGCAATGTCGTCACCTTCACCGGTAACGTGGTCGTG ACCCAGGGGACGATCAAGATTAACGCTGATAAAGTGGT GGTCACCGCGCCGGGCAACGAGAAAGGCAAAGAGGTG ATCGAAGGTTTCGGTAACCCGGCTACTTTCTATCAGATG CAGGATAACGGCAAGCCGGTAAAAGGCCGGGCGTCGA AAATGCGCTACGAGCTGCAGAACGATTACGTCGTGCTG ACGGGCAACGCTTATCTGGAACAGCTCGACAGCAACAT CAAGGGCGACAAGATCACCTATCTGGTGAAAGAGCAAA AAATGCAGGCCTTCAGCGACAAAGGTCGACGCGTCACC ACCGTTCTGGTGCCGTCCGAGCTGCAGGACAAAAGCGG CAACCAGCAAAAGAAGAGTAACTAATAAGGATCCAAA

Table S1. Primers and gBlocks used for the cloning of the protein constructs

**His$_6$-GB1-LptAm**	SGSHHHHHHSSGIEGRGRQYKLILNGKTLKGETTTEAVDAATAEKVFK QYANDNGVDGEWTYDDATKTFTVTESSGENLYFQ/GVTGDTDQPIHIES DQQSLDMQGNVVTFTGNVIVTQGTIKINADKVVVTRPGGEQGKEVIDG YGKPATFYQMQDNGKPVEGHASQMHYELAKDFVVLTGNAYLQQVDS NIKGDKITYLVKEQKMQAFSDKGKR
	GB1<br>TEV-site<br>LptAm<br>Mutations
***E. coli* sequences**	
**LptAm**<br>(LptA$_{28-159}$)	GVTGDTDQPIHIESDQQSLDMQGNVVTFTGNVIVTQGTIKINADKVVVT RPGGEQGKEVIDGYGKPATFYQMQDNGKPVEGHASQMHYELAKDFVV LTGNAYLQQVDSNIKGDKITYLVKEQKMQAFSDKGKR
**LptAm^{Q62L}**<br>(LptA$_{28-159}$ Q62L)	GVTGDTDQPIHIESDQQSLDMQGNVVTFTGNVIVTLGTIKINADKVVVT RPGGEQGKEVIDGYGKPATFYQMQDNGKPVEGHASQMHYELAKDFVV LTGNAYLQQVDSNIKGDKITYLVKEQKMQAFSDKGKR
**LptAm^{D33A}**<br>(LptA$_{28-159}$)	GVTGDTAQPIHIESDQQSLDMQGNVVTFTGNVIVTQGTIKINADKVVVT RPGGEQGKEVIDGYGKPATFYQMQDNGKPVEGHASQMHYELAKDFVV LTGNAYLQQVDSNIKGDKITYLVKEQKMQAFSDKGKR
**LptAm^{D33A,Q62L}**<br>(LptA$_{28-159}$ Q62L)	GVTGDTAQPIHIESDQQSLDMQGNVVTFTGNVIVTLGTIKINADKVVVT RPGGEQGKEVIDGYGKPATFYQMQDNGKPVEGHASQMHYELAKDFVV LTGNAYLQQVDSNIKGDKITYLVKEQKMQAFSDKGKR
**LptC^{AA}**<br>(LptC$_{25-191}$ Y60A R61A)	GAEKDDTAQVVVNNNDPTYKSEHTDTLVYNPEGALSAALIAQHVEYY SDQAVSWFTQPVLTTFDKDKIPTWSVKADKAKLTNDRMLYLYGHVEV NALVPDSQLRRITTDNAQINLVTQDVTSEDLVTLYGTTFNSSGLKMRGN LRSKNAELIEKVRTSYEIQNKQTQP
**mLptA**<br>(LptA$_{28-185}$ E39V M47A R76A)	GVTGDTDQPIHIVSDQQSLDAQGNVVTFTGNVIVTQGTIKINADKVVVT APGGEQGKEVIDGYGKPATFYQMQDNGKPVEGHASQMHYELAKDFVV LTGNAYLQQVDSNIKGDKITYLVKEQKMQAFSDKGKRVTTVLVPSQLQ DKNNKGQTPAQKKGN
**mLptA^{Q62L}**<br>(LptA$_{28-185}$ E39V M47A Q62L R76A)	GVTGDTDQPIHIVSDQQSLDAQGNVVTFTGNVIVTLGTIKINADKVVVT APGGEQGKEVIDGYGKPATFYQMQDNGKPVEGHASQMHYELAKDFVV LTGNAYLQQVDSNIKGDKITYLVKEQKMQAFSDKGKRVTTVLVPSQLQ DKNNKGQTPAQKKGN
***K. pneumoniae* sequences**	
**LptAm**<br>(LptA$_{28-159}$)	GKTGDTDQPIHIESDQQSLDMQGNVVTFTGNVVVTQGTIKINADKVVV TRPGNEKGKEVIEGFGNPATFYQMQDNGKPVKGRASKMRYELQNDYV VLTGNAYLEQLDSNIKGDKITYLVKEQKMQAFSDKGRR
**LptAm^{Q62L}**<br>(LptA$_{28-159}$ Q62L)	GKTGDTDQPIHIESDQQSLDMQGNVVTFTGNVVVTLGTIKINADKVVV TRPGNEKGKEVIEGFGNPATFYQMQDNGKPVKGRASKMRYELQNDYV VLTGNAYLEQLDSNIKGDKITYLVKEQKMQAFSDKGRR
**LptC^{AA}**<br>(LptC$_{25-191}$ Y60A R61A)	GANTDDTAQPEVNPNDPTYKSEHTDTVVYSPEGALSAALIAEHVEYFSD QEVSWFTKPVMTTFDTNKVPTWSVRADKAKLTNDRMLYLYGHVEVN ALAPDSQLRKITTDNAQINLVTQDVTSDDMVTLYGTTFNSSGLKMRGN LRSKNAELIEKVRTSYEIQNKQTQP
**mLptA**<br>(LptA$_{28-181}$ E39V M47A R76A)	GKTGDTDQPIHIVSDQQSLDAQGNVVTFTGNVVVTQGTIKINADKVVV TAPGNEKGKEVIEGFGNPATFYQMQDNGKPVKGRASKMRYELQNDYV VLTGNAYLEQLDSNIKGDKITYLVKEQKMQAFSDKGRRVTTVLVPSEL QDKSGNQQKKSN

Table S2. Amino Acid Sequences of Proteins.

Compound	Molecular formula	Monoisotopic mass (Da)	m/z found (ESI)	Mass found (ESI)	t_R in min†
thanatin	C103H177N35O27S3	2432.271	812.0 $[M+3H]^{3+}$	2433.0	2.99
1	C98H171N33O23S2	2242.267	748.4 $[M+3H]^{3+}$	2242.2	3.05
2	C103H174N32O24S2	2307.282	770.2 $[M+3H]^{3+}$	2307.6	2.79
3	C86H143N27O21S2	1954.0393	652.4 $[M+3H]^{3+}$	1954.2	2.92
4	C86H143N27O22S2	1970.034	657.7 $[M+3H]^{3+}$	1970.1	2.82
5	C84H139N25O22S2	1913.997	638.9 $[M+3H]^{3+}$	1913.7	2.72
6	C84H139N27O23S2	1957.998	653.7 $[M+3H]^{3+}$	1958.1	2.58
6-enant.	C84H139N27O23S2	1957.998	653.5 $[M+3H]^{3+}$	1957.5	2.55
7	C85H141N29O23S2	2000.020	667.7 $[M+3H]^{3+}$	2000.1	2.81
6-FL	C137H214N34O40S6	3167.408	1056.9 $[M+3H]^{3+}$	3167.7	3.08

Table S3. Analytical data of the synthesized peptides

†Analytical HPLC retention times (t_R, in minutes) were determined on Thermo Scientific Ultimate 3000RS, MS: Thermo Scientific MSQ plus using an Ascentis Express C8 column, 100 x 3 mm, 2.7 µm (Supelco, 53852-U) with the following solvents A (H_2O + 0.1% TFA) and B (CH_3CN + 0.085% TFA) and the gradient: 0-0.1 min: 95% A, 5% B; 7 min: 45% A, 55% B; 7.02-7.5 min: 3% A, 97% B; 7.52-7.8 min: 95% A, 5% B. Flow rate = 1.4 mL/min , column oven temperature 55°C, UV detection 220 nm, ionization method: ESI positive 60V profile mode.

A: ADMET properties.

Compound	Haemolysis	Cytotoxicity HeLa	Plasma protein binding (% bound)		Plasma Stability (% remaining 4 h)	
	% at 0.2 g/L	IC_{50} (μM)	Human	Mouse	Human	Mouse
**thanatin**	0	773	81	90	58	0
**1**	0	158	76	86	70	27
**2**	0	60	n.d.	n.d.	n.d.	89
**3**	0	41	n.d.	n.d.	n.d.	n.d.
**4**	0	536	91	86	100	98
**5**	0	1274	82	87	95	99
**6**	0	1161	62	89	92	90
**7**	0	456	81	94	97	97

B: PK parameters determined in plasma from male CD-1 mice after a single dose SC or IV injection.

Compound	Mouse PK SC		Mouse PK IV	
	C_{max}/D (ng/mL)*(mg/kg)	AUC/D (ng*h/mL) * (mg/kg)	C_0/D (ng/mL)*(mg/kg)	AUC/D (ng*h/mL)*(mg/kg)
**1**	55	49	n.d.	n.d.
**6**	486	747	1607	699
**7**	442	1172	2409	2117

C: Tolerability, 7-day repeated dosing and TK parameters determined in mice.

Compound	Tolerability (IV bolus, q12h)	7-day repeat dosing (IV bolus, q12h)	Mouse TK IV (top dose)	
			**C_0/D (ng/mL)*(mg/kg)**	**AUC/D (ng*h/mL)*(mg/kg)**
**6**	0/3 mice died at 40 mg/kg/day q12h	0/3 mice died at 30 mg/kg/day q12h	1340	941
**7**	0/3 mice died at 30 mg/kg/day q12h	0/3 mice died at 30 mg/kg/day q12h	1561	1640

Table S4. Biological profiling of thanatin and peptides 1-7.

<table>
<tr><th></th><th>Family</th><th>Species</th><th>LptA sequence identity</th><th>Thanatin</th><th>6</th><th>7</th></tr>
<tr><td rowspan="17">ENTEROBACTERALES</td><td rowspan="9">Enterobacteriaceae</td><td>Escherichia coli</td><td>100</td><td>1</td><td>0.03</td><td>0.06</td></tr>
<tr><td>Klebsiella oxytoca</td><td>100</td><td>2</td><td>0.25</td><td>0.5</td></tr>
<tr><td>Shigella flexneri</td><td>100</td><td>0.5</td><td>0.03</td><td>0.06</td></tr>
<tr><td>Citrobacter freundii</td><td>92</td><td>4</td><td>0.13</td><td>0.5</td></tr>
<tr><td>Salmonella enterica subsp. enterica</td><td>91</td><td>4</td><td>0.5</td><td>1</td></tr>
<tr><td>Enterobacter aerogenes</td><td>88</td><td>2</td><td>0.5</td><td>1</td></tr>
<tr><td>Klebsiella pneumoniae</td><td>88</td><td>1</td><td>0.13</td><td>0.25</td></tr>
<tr><td>Enterobacter cloacae</td><td>86</td><td>8</td><td>0.13</td><td>0.5</td></tr>
<tr><td>Raoultella planticola</td><td>84</td><td>2</td><td>0.06</td><td>0.25</td></tr>
<tr><td>Hafniaceae</td><td>Hafnia alvei</td><td>72</td><td>>8</td><td>2</td><td>4</td></tr>
<tr><td>Erwiniaceae</td><td>Pantoea agglomerans</td><td>69</td><td>>8</td><td>4</td><td>>8</td></tr>
<tr><td>Yersiniaceae</td><td>Serratia marcescens</td><td>64</td><td>>8</td><td>>8</td><td>>8</td></tr>
<tr><td rowspan="5">Morganellaceae</td><td>Morganella morganii</td><td>57</td><td>>8</td><td>>8</td><td>>8</td></tr>
<tr><td>Proteus vulgaris</td><td>55</td><td>>8</td><td>>8</td><td>>8</td></tr>
<tr><td>Providencia stuartii</td><td>55</td><td>>8</td><td>>8</td><td>>8</td></tr>
<tr><td>Proteus mirabilis</td><td>54</td><td>>8</td><td>>8</td><td>>8</td></tr>
<tr><td>Providencia rettgeri</td><td>54</td><td>>8</td><td>>8</td><td>>8</td></tr>
<tr><td colspan="2">Pseudomonadales</td><td>Moraxella catarrhalis</td><td>28</td><td>>8</td><td>>8</td><td>>8</td></tr>
<tr><td colspan="2">Xanthomonadales</td><td>Stenotrophomonas maltophilia</td><td>25</td><td>>8</td><td>>8</td><td>>8</td></tr>
</table>

Table S5. Thanatin activity spectrum versus LptA sequence conservation.
MIC values [µg/mL] determined by the CLSI microdilution method.

Family / species and number of isolates			Thanatin	6	7	Meropenem	Ciprofloxacin	Colistin
	MIC_{50}	(n=121)	2	0.25	0.25	>8	>8	0.13
	MIC_{90}	(n=121)	8	1	0.5	>64	>8	>8
MIC range*	Enterobacteriaceae (CS, Col-S)	(n=22)	1-4	0.06-1	0.06-2	≤0.06-0.5	≤0.01->8	0.06-0.5
	Enterobacteriaceae (CR, Col-S)	(n=85)	0.5-16	0.06-2	0.06-1	0.13->64	0.02->8	0.13-0.5
	Enterobacteriaceae (CS, Col- R)	(n=5)	1->8	0.06-2	0.06-1	0.06-1	≤0.01->8	2->8
	Enterobacteriaceae (CR, Col-R)	(n=9)	2-16	0.25-4	0.25-4	2->64	0.06->8	8->64
	E. coli ST131	(n=8)	1-2	0.06-0.25	0.13-0.25	<=0.06-8	0.02->8	0.13-0.25

Table S6. Minimum Inhibitory Concentrations (MIC values µg/mL) of thanatin and its derivatives compared to three standard of care antibiotics.

Abbreviations: Col-R: Colistin resistant; Col-S: Colistin sensitive; CR: Carbapenem resistant; CS: Carbapenem sensitive; MIC values [µg/mL] determined by the CLSI microdilution method; *MIC range: minimum and maximum MIC values for each panel.

Compound	*Escherichia coli* ATCC 25922			*Klebsiella pneumoniae* ATCC 43816		
	**4xMIC***	**8xMIC***	**16xMIC***	**4xMIC***	**8xMIC***	**16xMIC***
**Thanatin**	1.2E-06	2.0E-08	1.3E-08	n.t.	n.t.	n.t.
**6**	8.6E-09	<2.1E-09	<2.1E-09	2.9E-07	3.6E-08	5.0E-09
**7**	1.3E-07	4.8E-08	<3.7E-09	3.8E-08	3.1E-09	<3.1E-09
**5**	<3.7E-09	<3.7E-09	<3.7E-09	<3.1E-09	<3.1E-09	<3.1E-09

Table S7. Spontaneous frequency of resistance.

*MIC values of compounds on agarose plates. n.t.: not tested.

Strain	Mutant	LptA	Phenotype	Other genes	Function
Escherichia coli ATCC 25922	C244			kdsD (C48Y)	**Lipid A biosynthesis (KDO)**
	C245			kdsD (C48Y)	**Lipid A biosynthesis (KDO)**
	C250	E84D			**LPS transport**
	C292	Q62L			**LPS transport**
	C294	Q62L			**LPS transport**
	C296			purC (G65, SILENT)	Purine metabolism
	C298	Q62L			**LPS transport**
	C300	Q62L			**LPS transport**
	C493	Q62L			**LPS transport**
	C498	Q62L			**LPS transport**
	C504	Q62L			**LPS transport**
Klebsiella pneumoniae ATCC 43816	C261	L116Q			**LPS transport**
	C286	Q62L	small		**LPS transport**
	C287			hyp. Prot. 02520 (Y11D), dtpB (P441, SILENT)	Oligopeptide transporter
	C288		small	pglJ (T175 FRAME_SHIFT), phnJ (S247T), thiH (G121R)	Glycosylation, 2-aminoethylphosphonate-pyruvate transaminase, 2-iminoacetate synthase
	C289	Q62L		dtpB (P441, SILENT)	**LPS transport**, Oligopeptide transporter
	C290	I36N			**LPS transport**
	C307		mucoid	lapB (M1I)	**Lipid A biosynthesis regulation**
	C309	I36S			**LPS transport**
	C315	Q62L		dtpB (P441, SILENT)	Oligopeptide transporter
	C317	Q62L			**LPS transport**
Klebsiella pneumoniae NCTC 13443	C319	Q62L	small		**LPS transport**
	C333	Q62L			**LPS transport**
	C336	I36N		gsiD (P72T)	**LPS transport**, ABC transporter permease
	C339		small	lpxC (V37G), lpxD (Y80C)	**Lipid A biosynthesis**
	C355	I36S		fabF (A117V), mobA (S3I)	Fatty acid elongation, Molybdenum cofactor guanylyltransferase
	C356	Q62L		phnW (Q254 SILENT)	2-aminoethylphosphonate-pyruvate transaminase
	C363		small	yejM (GSRYL34V)	**Lipid A biosynthesis regulation**
	C367	Q62L			**LPS transport**
	C387	Q62L			**LPS transport**
	C405	I36S	small		**LPS transport**

Table S8. Mutations conferring resistance to thanatin, 4 or 6 identified by whole genome sequencing.

	Escherichia coli ATCC 25922				*Klebsiella pneumoniae* ATCC 43816							*Klebsiella pneumoniae* NCTC 13443				
**LptA mutation (occurrence in sequenced clones)**	**WT**	**E84D (1x)**	**Q62L (7x)**		**WT**	**L116Q (1x)**	**Q62L (4x)**	**I36N (1x)**	**I36S (1x)**			**WT**	**Q62L (5x)**	**I36S (2x)**		
**Other mutation (occurrence in sequenced clones)**				**KdsD (2x)**						**PglJ, PhnJ, ThiH (1x)**	**LapB (1x)**				**LpxC, LpxD (1x)**	**YejM (1x)**
**5**	0.125	1	0.25	0.5	0.25	1	4	1	1	0.5	0.5	1	8	4	0.5	2
**7**	0.125	1	2	0.5	0.25	2	8	2	2	0.5	0.5	1	>16	8	1	2
**Thanatin**	2	8	32	8	2	32	64	16	16	8	4	8	>64	32	4	16
**Meropenem**	≤0.06	≤0.06	≤0.06	0.13	0.13	≤0.06	≤0.06	≤0.06	≤0.06	0.13	0.13	>64	>64	>64	>64	>64
**Colistin**	0.25	0.25	0.13	0.25	0.13	0.13	0.13	≤0.06	0.13	0.13	0.13	0.25	0.13	0.13	0.25	0.25
**Ciprofloxacin**	≤0.01	≤0.01	≤0.01	≤0.01	0.03	0.03	0.03	0.03	0.03	0.03	0.03	>8	>8	>8	>8	>8
**Ceftazidime/Avi**	0.25	0.25	0.25	0.25	0.25	0.25	0.25	0.125	0.25	0.13	0.25	>64	>64	>64	>64	>64
**Novobiocin**	>64	>64	64	64	>64	>64	>64	>64	>64	32	32	>64	>64	>64	>64	>64
**Vancomycin**	>64	>64	>64	>64	>64	>64	>64	>64	>64	>64	>64	>64	>64	>64	>64	>64

Table S9. MIC values [µg/mL] of thanatin derivatives against a panel of strains harbouring LptA and other mutations.

Organism	Protein	Ligand	K_d ± 95CI (nM)
E. coli	LptAm	**6**-FL	6.9 ± 1.7
E. coli	$LptAm^{D33A}$	**6**-FL	13.8 ± 8.2
E. coli	$LptAm^{Q62L}$	**6**-FL	73.3 ± 21.3
E. coli	$LptAm^{Q62L, D33A}$	**6**-FL	82.4 ± 10.3
E. coli	mLptA	**6**-FL	968 ± 191
E. coli	$mLptA^{Q62L}$	**6**-FL	8140 ± 1180
K. pneumoniae	LptAm	**6**-FL	3.7 ± 0.9
K. pneumoniae	$LptAm^{Q62L}$	**6**-FL	242 ± 45.1
K. pneumoniae	mLptA	**6**-FL	453 ± 29.3

Table S10. Dissociation Constants for Protein-Peptide Interactions by FP (Direct Binding Assay)

Error ranges indicate the 95% confidence interval of the fit.

Organism	Protein	Competitor	IC_{50} ± 95CI (nM)	K_i ± 95CI (nM)
E. coli	LptAm	mLptA	212.6 ± 18.4	34.9 ± 3.0
E. coli	$LptAm^{Q62L}$	mLptA	101.9 ± 21.8	69.0 ± 14.7
E. coli	$LptAm^{Q62L}$	$mLptA^{Q62L}$	87.7 ± 13.7	59.3 ± 9.3
E. coli	LptAm	$LptC^{AA}$	10800 ± 2300	1800 ± 400
E. coli	$LptAm^{Q62L}$	$LptC^{AA}$	38700 ± 5100 *	26700 ± 3500 *
K. pneumoniae	LptAm	mLptA	145.4 ± 15.9	9.0 ± 1.0
K. pneumoniae	$LptAm^{Q62L}$	mLptA	73.2 ± 24.9	64.0 ± 21.8
K. pneumoniae	LptAm	$LptC^{AA}$	5120 ± 430	315.7 ± 26.4
K. pneumoniae	$LptAm^{Q62L}$	$LptC^{AA}$	7700 ± 4400 *	6800 ± 3900 *

Table S11. Inhibition Constants for Protein-Protein Interactions by FP (Indirect Assays)

* Extrapolated values for estimated affinity range for $LptAm^{Q62L}$ - $LptC^{AA}$ complexes
Error ranges indicate the 95% confidence interval of the fit.

Organism	Protein	Peptide	IC_{50} ± 95CI (nM)	K_i ± 95CI (nM)
E. coli	LptAm	thanatin	16.2 ± 1.0	2.7 ± 0.2
E. coli	LptAm	**1**	6.6 ± 0.5	1.1 ± 0.1
E. coli	LptAm	**2**	12.6 ± 0.7	2.1 ± 0.1
E. coli	LptAm	**5**	18.6 ± 1.1	3.1 ± 0.2
E. coli	LptAm	**6**	16.9 ± 1.0	2.8 ± 0.2
E. coli	LptAm	**7**	11.7 ± 0.5	1.9 ± 0.1
E. coli	$LptAm^{D33A}$	thanatin	14.3 ± 1.6	10.5 ± 1.5
E. coli	$LptAm^{D33A}$	**7**	32.3 ± 3.4	23.4 ± 2.5
E. coli	$LptAm^{Q62L}$	thanatin	50.0 ± 8.3	33.8 ± 5.6
E. coli	$LptAm^{Q62L}$	**1**	30.4 ± 8.8	20.5 ± 6.0
E. coli	$LptAm^{Q62L}$	**2**	19.3 ± 6.3	13.1 ± 4.2
E. coli	$LptAm^{Q62L}$	**5**	13.9 ± 2.3	9.4 ± 1.5
E. coli	$LptAm^{Q62L}$	**6**	14.6 ± 2.5	9.9 ± 1.7
E. coli	$LptAm^{Q62L}$	**7**	25.4 ± 4.6	17.2 ± 3.1
E. coli	$LptAm^{Q62L, D33A}$	thanatin	69.8 ± 19.8	49.2 ± 13.9
E. coli	$LptAm^{Q62L, D33A}$	**7**	23.2 ± 10.0	17.0 ± 7.3
K. pneumoniae	LptAm	thanatin	19.0 ± 0.8	1.8 ± 0.1
K. pneumoniae	LptAm	**1**	9.6 ± 0.5	0.9 ± 0.1
K. pneumoniae	LptAm	**2**	15.4 ± 0.8	1.5 ± 0.1
K. pneumoniae	LptAm	**5**	18.3 ± 0.8	1.8 ± 0.1
K. pneumoniae	LptAm	**6**	22.5 ± 1.1	2.2 ± 0.1
K. pneumoniae	LptAm	**7**	21.3 ± 1.0	2.0 ± 0.1
K. pneumoniae	$LptAm^{Q62L}$	thanatin	44.5 ± 9.0	38.9 ± 7.9
K. pneumoniae	$LptAm^{Q62L}$	**1**	20.8 ± 6.6	18.2 ± 5.8
K. pneumoniae	$LptAm^{Q62L}$	**2**	16.6 ± 4.7	14.5 ± 4.1
K. pneumoniae	$LptAm^{Q62L}$	**5**	25.7 ± 7.0	22.5 ± 6.1
K. pneumoniae	$LptAm^{Q62L}$	**6**	29.0 ± 8.8	25.3 ± 7.7
K. pneumoniae	$LptAm^{Q62L}$	**7**	18.4 ± 3.9	16.1 ± 3.4

Table S12. Inhibition Constants for Protein-Peptide Interactions by FP (Indirect Assays)
Error ranges indicate the 95% confidence interval of the fit.

	E. coli LptAm-**7**	*E. coli* LptAm^{Q62L}-**7**	*K. pneumoniae* LptAm-**7**	*E. coli* LptAm^{Q62L}-**5**
**pdb code**	7QS6	7ZED	7ZAX	8BSS
**bmrb code**	34699	34720	34716	34773
**NOE distance restraints**				
Total	1186	1036	1686	1103
Intra-residue, i-j=0	285	277	425	313
Sequential, i-j=1	383	335	439	365
Medium-range, 1<i-j<5	98	68	139	81
Long-range, i-j ≥ 5	339	328	683	344
Intermolecular	81	29	68	55
Torsion angle constraints	182	170	186	206
**Structure statistics (20 conformers)**				
CYANA target function value (Å^2)	6.3	3	3.2	3.6
**Satisfaction of Experimental Constraints**				
Distance constraint violation				
Number >0.2 A	18 ± 5	7 ± 2	8 ± 2	12 ±3
Maximum (**Å)**	0.44 ± 0.11	0.22 ± 0.03	0.32 ± 0.09	0.37±0.09
Torsion angle constraint violations				
Number >5°	6 ± 3	0	0	0
Maximum (deg)	19.7 ± 1.2	1.6 ± 0.25	1.67 ± 0.26	2.62 ± 0.94
PROCHECK Ramachandran plot analysis				
Residues in favoured regions (%)	74.2	80.2	78.1	78.5
Residues in additional allowed regions (%)	23.6	18.4	21.0	19.9
Residues in generously allowed regions (%)	2.0	0.5	0.9	1.4
Residues in disallowed regions (%)	0.2	0.9	0	0.1
**RMSD to the average coordinates (Å)**				
Backbone atoms (residues 28-145,6-21)	1.08 ± 0.25	1.61 ± 0.38	1.34 ± 0.26	1.84 ± 0.40
Heavy atoms (residues 28-145,6-21)	1.56 ± 0.25	1.99 ± 0.29	1.73 ± 0.22	2.15 ± 0.33
Backbone atoms (regular secondary structure)[a]	0.68 ± 0.15	0.62 ± 0.11	0.56 ± 0.09	0.77 ± 0.11
Heavy atoms (regular secondary structure)[a]	1.17± 0.17	1.10 ± 0.11	0.96 ± 0.10	1.25 ± 0.18

[a] protein residues 37-45, 52-62, 65-75, 85-97, 105-115, 120-130, 135-143 and compound **7** 10-12, 18-19

Table S13. Statistics from the NMR structure calculations of the LptAm and LptAm^{Q62L} complexes.
